# Tumor cell senescence-induced macrophage CD73 expression is a critical metabolic immune checkpoint in the aging tumor microenvironment

**DOI:** 10.7150/thno.91119

**Published:** 2024-01-20

**Authors:** Yue Deng, Qinyan Chen, Xiao Yang, Yajie Sun, Bin Zhang, Wenwen Wei, Suke Deng, Jingshu Meng, Yan Hu, Yijun Wang, Zhanjie Zhang, Lu Wen, Fang Huang, Chao Wan, Kunyu Yang

**Affiliations:** 1 Cancer Center, Union Hospital, Tongji Medical College, Huazhong University of Science and Technology, Wuhan 430022, China; 2 Institute of Radiation Oncology, Union Hospital, Tongji Medical College, Huazhong University of Science and Technology, Wuhan 430022, China; 3 Hubei Key Laboratory of Precision Radiation Oncology, Wuhan 430022, China

**Keywords:** senescence, CD73, tumor-associated macrophages, cancer immunotherapy, tumor microenvironment

## Abstract

**Background:** The role of senescent cells in the tumor microenvironment (TME) is usually bilateral, and diverse therapeutic approaches, such as radiotherapy and chemotherapy, can induce cellular senescence. Cellular interactions are widespread in the TME, and tumor cells reprogram immune cells metabolically by producing metabolites. However, how senescent cells remodel the metabolism of TME remains unclear. This study aimed to explore precise targets to enhance senescent cells-induced anti-tumor immunity from a metabolic perspective.

**Methods:** The *in vivo* senescence model was induced by 8 Gy×3 radiotherapy or cisplatin chemotherapy, and the *in vitro* model was induced by 10 Gy-irradiation or cisplatin treatment. Metabonomic analysis and ELISA assay on tumor interstitial fluid were performed for metabolites screening. Marker expression and immune cell infiltration in the TME were analyzed by flow cytometry. Cell co-culture system and senescence-conditioned medium were used for crosstalk validation *in vitro*. RNA sequencing and rescue experiments were conducted for mechanism excavation. Immunofluorescence staining and single-cell transcriptome profiling analysis were performed for clinical validation.

**Results:** We innovatively reveal the metabolic landscape of the senescent TME, characterized with the elevation of adenosine. It is attributed to the senescent tumor cell-induced CD73 upregulation of tumor-associated macrophages (TAMs). CD73 expression in TAMs is evoked by SASP-related pro-inflammatory cytokines, especially IL-6, and regulated by JAK/STAT3 pathway. Consistently, a positive correlation between tumor cells senescence and TAMs CD73 expression is identified in lung cancer clinical specimens and databases. Lastly, blocking CD73 in a senescent background suppresses tumors and activates CD8^+^ T cell-mediated antitumor immunity.

**Conclusions:** TAMs expressed CD73 contributes significantly to the adenosine accumulation in the senescent TME, suggesting targeting CD73 is a novel synergistic anti-tumor strategy in the aging microenvironment.

## Introduction

Senescence, a cellular stress response triggered by molecular damage, has been listed as one of the hallmarks of cancer 1. Numerous antitumor treatments can cause cellular senescence, including radiotherapy, chemotherapy, and targeted therapies 2, 3. Besides morphological, transcriptional level, epigenetic and metabolic alterations, the most prominent characteristic of senescent cells is the generation of senescence-associated secretory phenotypes (SASP) 4. Senescent cells in the tumor microenvironment (TME) often have pleiotropic roles in promoting immune cell recruitment, wound healing, and cell plasticity/stemness, while on the other hand, senescent cells contribute to paracrine senescence, immune evasion, and tissue dysfunction/inflammation 5. Thus, their impact on tumor progression is also bidirectional. Although senescent cell clearance has been shown effectively to reverse the progression of multiple age-related pathologies in previous preclinical studies 6, given that it is inherently a double-edged sword, exploring more precise molecular targets or pathways that contribute to the detrimental effects of senescent cells is particularly important to maximize the balance of senescent cells in favor of the anti-tumor side.

Interleukin 6 (IL-6) is involved in the immunomodulation of a wide range of diseases and has context-dependent pro- and anti-inflammatory properties. Innovative therapeutic approaches targeting the IL-6 pathway are currently employed in the treatment of many rheumatic diseases 7-10. In the TME, abundant IL-6 can directly stimulate tumor cells proliferation and survival, and also induce the secretion of pro-inflammatory and pro-angiogenic factors 11. IL-6 is produced by tumor cells, stromal cells and immune cells infiltrated in the TME. Thus, tumor-associated macrophages (TAMs) are also a major source of IL-6, especially M1 macrophages 11, 12. It has been shown that the major components of the SASP are proinflammatory cytokines IL-6 and IL-8 4, 13. The effects of IL-6 in senescence are also multifaceted. As beneficial functions, senescent cells promote senescence in neighboring cells by secreting IL-6 and IL-8, limiting tumor development in a cell non-autonomous manner. As deleterious functions, IL-6 participates in promoting chronic inflammatory microenvironment, thereby supporting tumor development. In addition, senescent cells promote reprogramming of the embryonic state partially through IL-6, which can support tissue regeneration on the one hand and facilitate tumor development on the other 4, 13-15. However, it has not been fully illustrated how IL-6 regulates TAMs in the senescent microenvironment.

There are extensive interactions between tumor cells and immune cells in the TME 16, and increased evidence suggests that tumor cells suppress the function of immune cells such as tumor-infiltrating T cells by competing and consuming nutrients such as glucose, amino acids, and glutamine 17-19. Besides nutrient consumption, metabolites such as lactate, PGE2, and arginine produced by tumor cells have a profound impact on immune cells in the TME, altering the metabolic adaptations of immune cells and contributing to their metabolic reprogramming 20-24. However, whether senescent tumor cells have an effect on the metabolism of TME and how they reprogram the microenvironmental metabolism has not been elucidated yet.

The cell surface ectonucleotidases CD73 synergizes with CD39 to hydrolyze extracellular ATP to adenosine 25. The adenosine accumulation in TME has been reported to inhibit the anti-tumor function of various immune cells, including NK cells and cytotoxic T cells 26, 27. Here, we innovatively reveal the metabolic landscape of the senescent TME, characterized with the elevation of adenosine. We demonstrate that senescent tumor cells increased the CD73 expression in TAMs, which substantially contributes to the adenosine accumulation in TME. It is induced by SASP-related pro-inflammatory cytokines, in particular IL-6, and regulated by JAK/STAT3 signaling pathway *in vitro*. Finally, we elucidate that blocking CD73 enhances CD8^+^ T cell-mediated antitumor immunity. Hence CD73 may serve as a critical metabolic brake for immunotherapeutic target in the aging background.

## Materials and Methods

**Human specimens**: Human specimens were acquired with the approval of the authors' institute. Written consent was obtained from the participants prior to the study.

**Chemical reagents**: ABT263 (S1001), LY-3475070 (S9867), BP-1-102 (S7769), TPCA1 (S2824), Ruxolitinib (S1378) were purchased from Selleck. Recombinant Murine IL-1α (211-11A), Recombinant Murine IL-1β (211-11B), Recombinant Murine IL-6 (216-16), Recombinant Murine IL-10 (210-10), Recombinant Murine TNF-α (315-01A) were purchased from PeproTech. Recombinant Mouse IFN-α (752802) was purchased from BioLegend.

**Cell lines and cell culture**: LLC, B16-F10, MC38, SCC7, and Hepa1-6 cell lines were obtained from the American Tissue Culture Collection (ATCC). LLC, MC38, SCC7, and Hepa1-6 were cultured in Dulbecco's Modified Eagle's Medium (DMEM) (Gibco, Grand Island, NY, USA), while B16-F10 was maintained in RPMI 1640 supplemented with 10% Fetal Bovine Serum (FBS) (Gibco, Grand Island, NY, USA) and 1% penicillin/streptomycin solution at 37 °C with 5% CO_2_.

**Generation of BMDMs**: BMDMs were generated from femurs of 6- to 12-week C57BL/6 male mice. After removal of RBCs using RBC lysis buffer, BMDMs were differentiated in RPMI 1640 with 10% FBS and macrophage colony-stimulating factor (20 ng/mL; PeproTech). The naïve macrophages (BMDMs) were obtained on the sixth day.

**T-Cell Proliferation and Cytokine Secretion Assays**: CD8^+^ T cells were isolated from the spleens of C57BL/6 mice according to the protocol of MojoSort™ Mouse CD8^+^ T Cell Isolation Kit (480008, Biolegend). BMDMs were initially treated with LLC CM for 24 h. Then replace the fresh medium and continue the incubation for 2 h before collecting the supernatant of BMDMs. Subsequently, the BMDMs CM was filtered through a 0.22 μm filter. To investigate the effect of macrophages on T cell function, BMDMs were treated with 1 μM LY-3475070 (CD73 inhibitor) in LLC CM for 24 h, and then BMDMs CM was collected for T cell culture as mentioned previously. Carboxyfluorescein succinimidyl ester (CFSE)-labeled CD8^+^ T cells were cultured for 4 days with CM containing IL-2 (20 ng/mL) and Dynabeads® Mouse T-Activator CD3/CD28 (11452D, Life Technologies). Finally, the proliferation and GrzB, IFN-γ secretion were detected by flow cytometry.

**Radiation**: LLCs were irradiated by single doses of 10 Gy. For subcutaneous tumors, mice were anesthetized and the right posterior limbs (with tumors) were exposed to a single dose of 8 Gy radiation. The standard parameters for dose delivery were beam quality of 6 MV and dose rate of 6 Gy/min. The radiation doses were confirmed by thermo-luminescent dosimeters (TLDs).

**SA-β-Gal staining assay**: Fresh frozen sections of tumor tissue, or indicated cells were fixed with 4% paraformaldehyde (Servicebio) for 15 min, and then stained with a Senescence β-Galactosidase Staining Kit (C0602, Beyotime) according to the manufacturer's instructions.

**ELISA**: The adenosine concentration of TIF, and the concentrations of adenosine, IL-6, IL-1β, CXCL10, TNFα and CCL5 in medium supernatants were measured by ELISA (FANKEW, F9383-A; Proteintech, KE10007; DAKEWE, 1210122; ABclonal, RK00056, RK00027, RK00167) according to the manufacturer's instructions.

**Conditioned medium (CM)**: To obtain CM, LLCs exposed to 10 Gy radiation were cultured in DMEM with 10% FBS for two days and replaced with serum-free DMEM on the third day. The serum-free medium was then collected after 24 h of incubation and filtered with a 0.22 μm filter (MilliporeSigma). When used for BMDMs culture, 10% FBS was added to generate CM. To neutralize IL-6 in it, CM was added with 10 μg/mL anti-IL-6-neutralizing antibody (BE0046, BioXCell) and incubated for 4 h at 37 °C before being used for cell culture.

**Transfections**: BMDMs seeded in 6-well plates were transfected with either small interfering RNA (siRNA) targeting STAT3 or negative control (NC) siRNA (Genecreate Biological Engineering CO) using Lipofectamine RNAiMAX (Invitrogen, Carlsbad, CA, USA). Cells were harvested and processed for qPCR 48 h later. Short hairpin RNA (shRNA) against p65 (shp65) and a negative control shRNA (shNC) were designed and synthesized by Vigene (Vigene Biology, Shandong, China). For transient transfection, Lipofectamine 2000 reagent (Invitrogen, Carlsbad, CA) was used when the LLC cell fusion was 60-70%. Transfected cells were screened with 2 μg/mL puromycin for 5 days and then knockdown efficiency was confirmed by RT-qPCR. The RNA sequences used for transfection in this study are listed in **Table S1 and S2**.

**Western blotting**: Cells were lysed with RIPA lysis buffer containing protease inhibitors and phosphatase inhibitor at 4 °C for 30 min. After centrifugation at 12,000g for 30 min at 4 °C, protein was acquired and quantified using the BCA Protein Assay Kit (Thermo Scientific). Samples were then mixed with SDS-polyacrylamide gel electrophoresis sample loading buffer (P0015, Bain-marie Biotech) and denatured at 100 °C for 10 min. They were then separated in an SDS-polyacrylamide gel and transferred to polyvinylidene difluoride (PVDF) membranes. PVDF membranes were blocked at room temperature with 5% skim milk for 1 h, followed by overnight incubation with primary antibodies at 4 °C. Next, membranes were incubated with secondary antibody for 1 h at room temperature. The blots were exposed to chemiluminescence using NcmECL Ultra (P10100, NCM Biotech). The antibodies used are listed in **Table S3**.

**Real-time Quantitative PCR**: Total RNA was extracted using Total RNA Kit I R6834 (Omega) and reverse-transcribed into complementary DNA using HiScript III RT SuperMix (+ gDNA wiper) (#R323, Vazyme) according to the manufacturer's instructions. The RT-qPCR reaction was executed in a StepOnePlus Real-Time PCR System (Thermo Fisher Scientific) using the AceQ® Universal SYBR qPCR Master Mix (Vazyme). Primer sequences are listed in **Table S4** and are synthesized by Sangon Biotech Co., Ltd (Shanghai).

**Immunofluorescence staining**: Tumor tissues were fixed in 4% paraformaldehyde, paraffin-embedded, and cut into 3μm sections. For immunofluorescence staining, sections were de-paraffinized, rehydrated, and boiled in 10 mM citrate buffer (pH 6.0) for 15 min in a microwave oven for antigen retrieval. Antibodies were incubated overnight at 4 °C. The primary antibodies were listed: F4/80 (70076, CST), CD73 (12231-1-AP, Proteintech), CDKN2A (ab189034, Abcam), CD68 (66231-2-Ig, Proteintech). Secondary Alexa Fluor 488 or 594 dye-conjugated antibodies (Servicebio, 1:200) were applied for 1 h, and nuclei were stained with DAPI (G1012, Servicebio, 1:200) for 10 min at room temperature. Immunofluorescence slides were visualized with a confocal fluorescence microscope (Dragonfly/CR-DFLY-201-40).

**RNA sequencing**: BMDMs were collected after co-culture with senescent or control LLCs, and sent to Beijing Novogene Technology Co., Ltd. for RNA sequencing analysis.

**LC-MS/MS Analysis**: TIF of the senescent and senescence-clearing LLC subcutaneous tumors was evaluated by metabonomic analysis. The TIF in senescence group was colloected one week after receiving 8 Gy×3 radiotherapy, while the TIF in the senescence-clearing group was collected after five consecutive days of ABT263 treatment following 8 Gy×3 radiotherapy. Samples were stored at -80 °C and sent to Wuhan Metware Biotechnology Co., Ltd. for subsequent LC-MS/MS analysis.

**Mouse Tumor Models and Therapeutic effect evaluation**: Female C57BL/6 mice (6-week-old) were purchased from Wuhan Shubeili Biology Science and Technology Co., Ltd. All mice were raised in compliance with the protocols approved by the Animal Experimentation Ethics Committee of the Huazhong University of Science and Technology (HUST, Wuhan, China).

To establish the subcutaneous tumor-bearing model, 100 μL PBS containing 1×10^6^ LLC cells was subcutaneously injected into the right flank of mice. Two weeks after LLC inoculation, mice were divided randomly into different groups. Treatment was initiated when the tumor volume reached 50 mm^3^. In the radiotherapy-induced aging model, 8 Gy×3 radiotherapy was given to subcutaneous tumors. In the chemotherapy-induced aging model, 100 mg/kg cisplatin was given by a single intraperitoneal injection. ABT263 (50 mg/kg) or LY-3475070 (50 mg/kg) dissolved in a solution (5% DMSO + 95% Corn oil) was administered by gavage once a day for five days after radiotherapy or chemotherapy. Anti-PD-1 mAb (10 mg/kg) was administered intraperitoneally every other day, for a total of three times. The tumor size was measured every two days. The mice were sacrificed when the tumor size reached 1000 mm^3^.

**Flow Cytometry**: To analyze immune cell infiltration in TME, subcutaneous tumors were excised and digested with Hyaluronidase and Collagenase V. Tumor tissues were grinded into single cell suspensions, followed by lysis of RBCs and resuspension in PBS. For cell surface antigen analysis, cells were first stained with Zombie NIR™ Fixable Viability Kit (Biolegend, 423106), Zombie Violet™ Fixable Viability Kit (Biolegend, 423114) to remove dead cells, and then stained with the anti-mouse αCD39 (Biolegend, 143804), αCD73 (Biolegend, 127232), αCD45 (Biolegend, 103132; 157214), αCD3 (Biolegend, 100204), αCD4 (Biolegend, 100422), αCD8a (Biolegend, 100751), αCD11b (Biolegend, 101228), αF4/80 (Biolegend, 123114), αPD1 (Biolegend, 135218), αCTLA4 (Biolegend, 106306), αLAG3 (Biolegend, 125210) in recommended concentrations and incubated at 4 °C for 30 min. For intracellular IFN-γ (Biolegend, 505830), GrzB (Biolegend, 372208), and Foxp3 (Thermofisher, 17577382) cytokine staining in T cells, cells were fixed and permeabilized after stimulation with Monensin sodium salt (ab120499, Abcam, 1 ug/mL), Ionomycin calcium salt (5608212, PeproTech, 100 ng/mL), and Phorbol 12-myristate 13-acetate (PMA) (ab120297, Abcam, 100 ng/mL) for 5 h at 37 °C with 5% CO_2_. For the CD86 (Biolegend, 105012) and CD206 (Biolegend, 141706) staining, cells were also fixed and permeabilized.

**Macrophage depletion**: To delete macrophages, clodronate liposomes-Anionic (FormuMax, F70101C-A-10) was injected intraperitoneally every three days for a total of three times. The first dose was 200 μL of each, and the second two doses were 150 μL of each.

**TCGA Database Analysis**: The raw RNA-seq data of lung adenocarcinoma were obtained from the TCGA database (https://gdc.nci.nih.gov/). Immune infiltration and gene expression correlation were analyzed using the Xiantao Academic Online Tools (https://www.xiantao.love/products).

**Single-cell RNA sequencing (scRNA-seq) analysis**: The scRNA-seq data of metastatic lung adenocarcinoma 28 was acquired from GSE131907. Before further analysis, quality measures were performed on raw gene-cell-barcode matrix for each cell: mitochondrial genes (≤ 20%), unique molecular identifiers (UMIs), and gene count (ranging from 100 to 150,000 and 200 to 10,000). The UMI count for the genes in each cell was log-normalized to transcripts per million (TPM)-like values. For each batch, genes with low expression levels (min.cells < 0.1%) were removed. Relative expression levels across the remaining subset of cells and genes were centered. Unsupervised dimensional reduction and clustering were conducted. Variably expressed genes for principal components (PCs) calculation were selected, with a cutoff value of quantile-normalized variance > 0.5 and mean expression between 0.0125 and 3. Significant PCs subset selection and cell clustering were performed using Seurat package v2.3.4. UMAP visualization was applied using UMAP package. The cell cluster annotations were defined by the expression levels of known cellular markers. All analysis was performed with R version 3.4.

**Statistical analysis**: The statistical analysis involved was performed with GraphPad Prism 8.0. For comparison of two groups, the unpaired two-tailed Student's t-test was used. For comparisons of three or more groups, one-way analysis of variance (ANOVA) and Tukey's multiple comparisons test were performed. Survival curves were evaluated with the log-rank (Mantel-Cox) test, while the tumor growth was compared by two-way ANOVA and Tukey's multiple comparisons test. Flow cytometry data were analyzed by Flowjo.10.8.1. Data are presented as mean ± SEM. Significant differences between the groups are indicated by *p <0.05; **p < 0.01; ***p < 0.001; and ns, not statistically significant.

## Results

### Adenosine is abundant in the senescent microenvironment

**Figure 1:**
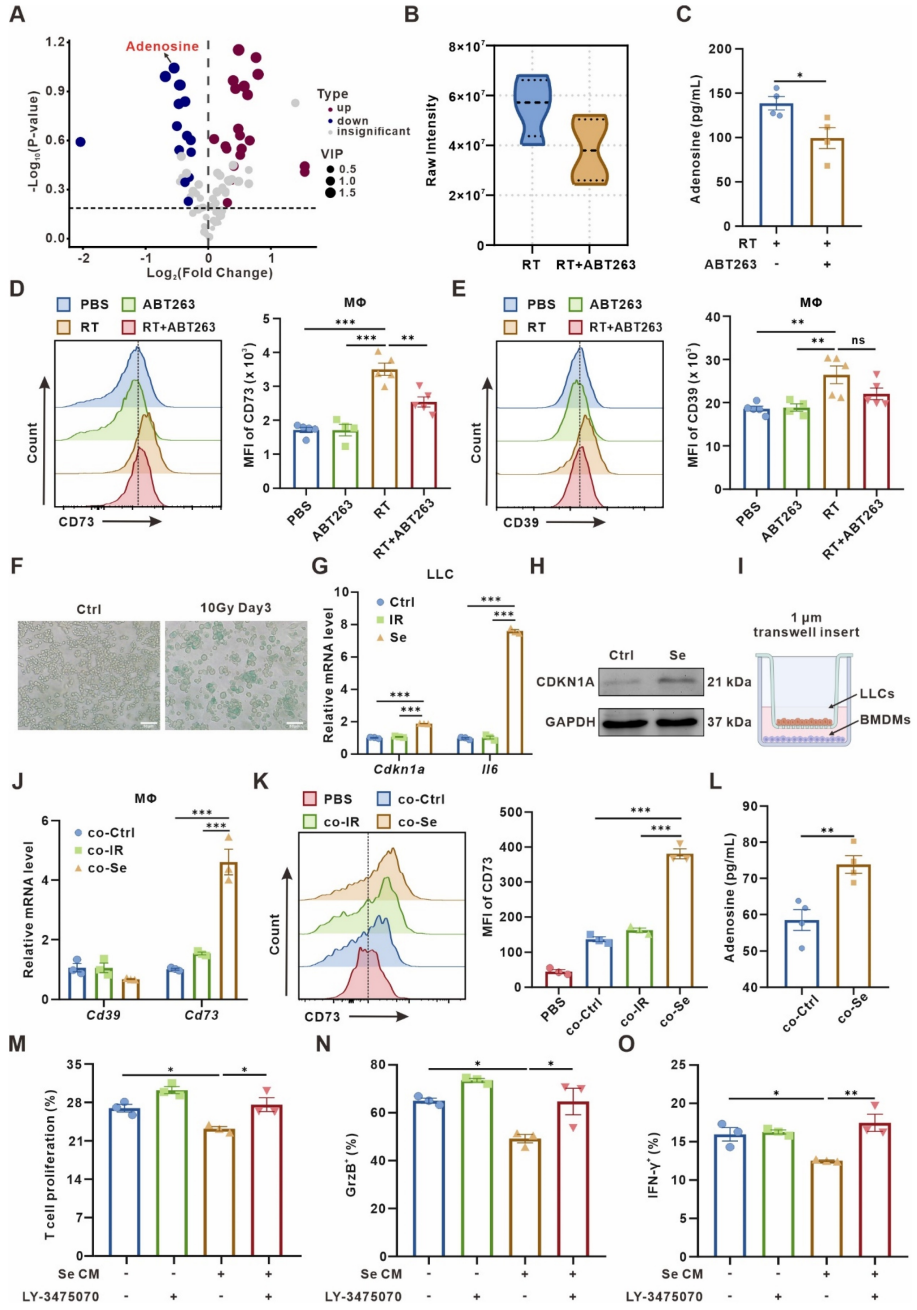
** Senescent tumor cells induce adenosine-rich TME by promoting upregulation of macrophage CD73 expression. (A)** Volcano plot showing the upregulated, downregulated, or insignificantly differential nucleotide-related metabolites in the senescence-clearing (RT+ABT263) TIF compared to the senescence (RT) TIF. **(B)** Adenosine content in senescence (RT) and senescence-clearing (RT+ABT263) TIF detected by LC-MS/MS. **(C)** Adenosine content in RT and RT+ABT263 TIF detected by ELISA. (**D-E**) Left panel, Flow cytometric analysis of CD73 **(D)** or CD39 **(E)** expression on TAMs in LLC subcutaneous tumor under various treatment conditions (n = 4 to 5 per group). Right panel, statistical analysis of CD73 **(D)** or CD39 **(E)** expression. The indicated results represent the mean ± SEM, analyzed by one-way ANOVA. MFI, mean fluorescence intensity. **(F)** LLC murine lung cancer cells senescence detected by SA-β-Gal staining assay. Scale bar: 50 μm. **(G)** Relative mRNA expression of senescence-associated genes in LLCs three days after 10 Gy-radiation. The indicated results represent the mean ± SEM of 3 independent experiments. **(H)** Western blot identifying the expression changes of CDKN1A in LLCs three days after 10 Gy-radiation. **(I)** Co-culture pattern diagram of tumor cells and BMDMs. **(J)** Relative mRNA expression of *Cd39* and *Cd73* in BMDMs after co-culture with Ctrl LLCs (Ctrl), LLCs one day after 10 Gy-radiation (IR), or LLCs three days after 10 Gy-radiation (Se) for 24 h. The indicated results represent the mean ± SEM of 3 independent experiments. **(K)** Left panel, Flow cytometric analysis of CD73 expression on BMDMs after co-culture with LLCs as described in **(J)** for 24 h. Right panel, statistical analysis of CD73 expression. The indicated results represent the mean ± SEM of 3 independent experiments, analyzed by one-way ANOVA. **(L)** Adenosine content in the supernatant of BMDMs detected by ELISA after co-culture with control (Ctrl) or senescent (Se) LLCs for 24 h. **(M)** CD8^+^ T cells were labeled with CFSE dye prior to co-culture, and the proportion of proliferative T cells with low CFSE expression was detected by flow cytometry after incubation with conditioned medium as indicated in different groups for 72 h. The indicated results represent the mean ± SEM of 3 independent experiments, analyzed by one-way ANOVA. (N-O) After incubation with different conditioned medium for 72 h, the percentage of GrzB^+^CD8^+^ T cells in CD8^+^ T cells **(N)** and the percentage of IFN-γ^+^CD8^+^ T cells in CD8^+^ T cells **(O)** in each group were detected by flow cytometry. The indicated results represent the mean ± SEM of 3 independent experiments, analyzed by one-way ANOVA. *p < 0.05; **p < 0.01; ***p < 0.001; ns, not statistically significant.

To identify the metabolic alterations in senescent TME, we used 8 Gy×3 radiotherapy to induce senescence in a subcutaneous murine LLC lung cancer model, which was confirmed successfully constructed by testing senescence-associated β-galactosidase (SA-β-gal) activity **(Figure S1A)**. Additionally, ABT263, a widely recognized senolytic 29, was administered by gavage to mice to clear senescent cells. Herein, metabonomic analysis was conducted on tumor interstitial fluid (TIF) of the senescent (RT) and senescence-clearing (RT+ABT263) LLC tumor-bearing mice by liquid chromatography-tandem mass spectrometry (LC-MS/MS). We found 526 differential metabolites including amino acids, organic acids, FA, nucleotides, and their metabolites **(Figure S1B, Table S5)**. As nucleotides are essential components for cell proliferation and functions 30, we focused on the differential nucleotides and their metabolites in the senescent TME. Forty-one differential nucleotide-related metabolites were filtered, of which the significant down-regulation of adenosine in the senescence-clearing group caught our attention **(Figure 1A-B)**. The ELISA assay also confirmed that the TIF of senescence group contained more adenosine** (Figure 1C)**.

The ectonucleotidases CD39 and CD73 on the cytomembrane can convert extracellular ATP and ADP to immunosuppressive adenosine through a cascade reaction 25. To investigate the main sources of adenosine in the senescent microenvironment, we examined CD39 and CD73 expression in major immune cell populations in LLC subcutaneous tumor by flow cytometry. It indicated that CD73 and CD39 was significantly upregulated on macrophages in the senescence group compared to the PBS group, while CD73 was downregulated on macrophages after combined with ABT263 **(Figure 1D-E)**. We examined CD73 expression in the senescence group and found it predominantly expressed in CD86^+^ M1 macrophages **(Figure S1C-E)**. In contrast, CD39 and CD73 expression on other immune cells, such as CD4^+^ and CD8^+^ T cells, was not remarkably altered after senescence clearance **(Figure S1F)**. These findings suggested that adenosine accumulation in the senescent TME is catalyzed by CD73 on the macrophages.

### Senescent tumor cells facilitate macrophages CD73 expression *in vitro*

In senescent TME, CD73 expression was markedly upregulated in macrophages *in vivo*, but not elevated *in vitro* by direct 10 Gy-irradiation on bone marrow-derived macrophages (BMDMs) **(Figure S2A)**. Previously, the crosstalk between senescent tumor cells and others was reported to influence cellular function and remodel the TME 16. We detected significant expression of senescence-associated markers in LLC cells on the third day after 10 Gy-irradiation *in vitro*, such as SA-β-Gal activity, *Cdkn1a* and *Il6* **(Figure 1F-H)**. We next used a cell co-culture system as shown in **Figure 1I**, using a 1 μm transwell insert with senescent or control LLC cells in the upper chamber and BMDMs in the lower chamber **(Figure 1I)**, and the expression of CD39 and CD73 in BMDMs was assayed after co-culturing for 24 h. Both qPCR and flow cytometry indicated a remarkable upregulation of CD73 in BMDMs after co-culture with senescent LLC, while CD39 expression showed no alteration **(Figure 1J-K)**. Moreover, the adenosine in the culture supernatant of BMDMs after senescence co-culture was also significantly elevated **(Figure 1L)**. We also conducted co-culture experiments in B16-F10 murine melanoma cells, MC38 murine colon carcinoma cells, SCC7 murine squamous cell carcinoma cells, and Hepa1-6 murine hepatoma cells, respectively, which showed an consistent upregulation of CD73 in BMDMs co-culturing with senescent tumor cells **(Figure S2B)**. In contrast, co-culture of the lower chamber cells with bone marrow-derived dendritic cells (BMDCs) revealed no alteration on CD73 expression in BMDCs **(Figure S2C)**. Chemotherapy can also induce cellular senescence, and we demonstrated that LLC cells treated with 0.5 or 1.0 μg/mL cisplatin for 24 h and then withdrawn showed senescent characteristics on the third day **(Figure S2D-F)**. Cisplatin-induced senescent LLC cells could also promote CD73 expression in BMDMs, but cisplatin failed to upregulate CD73 directly **(Figure S2G-H)**. These results suggested that senescent tumor cells boost CD73 expression in macrophages *in vitro*, promoting adenosine production.

To investigate whether macrophages are the main “CD73 producer” in the TME, we used Clodronate liposomes-Anionic (Clo) to scavenge macrophages in mice. The clearance efficiency of macrophages in the spleen and peripheral blood of mice was over 70% **(Figure S2I-J)**. We found that the clearance of macrophages significantly reversed senescence-induced CD73 elevation *in vivo* **(Figure S2K)**, indicating that macrophages-expressed CD73 accounts for an important proportion in the senescent TME.

As T cells are the mainstay exerting anti-tumor immunity, we wondered how senescence-remodeled macrophages affect T cells function. After being remodeled by senescent tumor cells for 24 h, we collected the conditioned medium from BMDMs (named as Se CM) for CD8^+^ T cells culture. The sorted mouse spleen CD8^+^ T cells were incubated with Se CM for 72 h, and the proliferation of T cells and the secretion capacity of Granzyme B (GrzB) and IFN-γ were measured. It indicated that macrophages co-cultured with senescent tumor cells significantly suppressed the proliferation and activity of T cells, which could be rescued by LY-3475070, CD73 inhibitor, on BMDMs **(Figure 1M-O, Figure S3)**.

### SASP cytokines contribute to CD73 expression in macrophages

**Figure 2:**
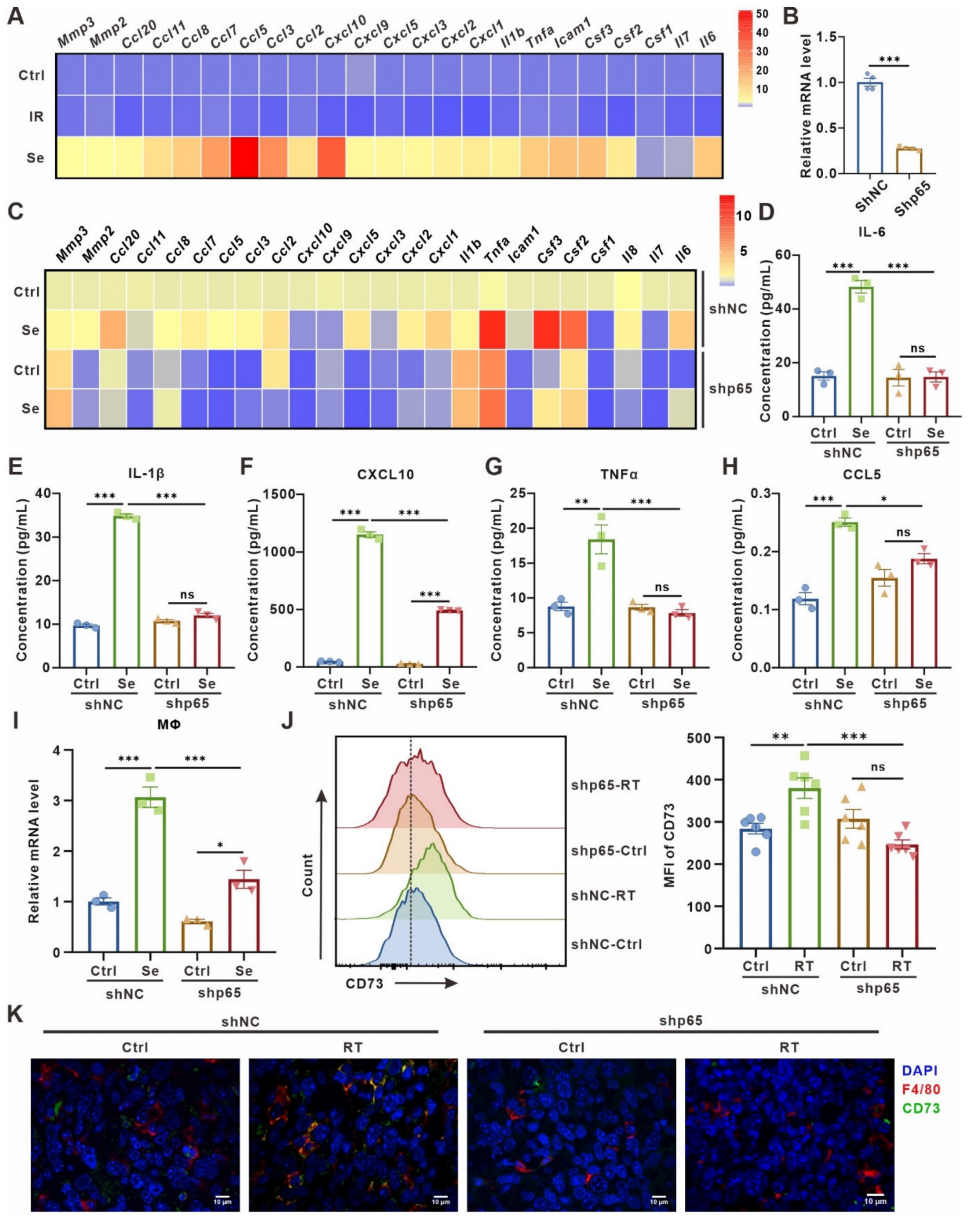
** SASP cytokines contribute to CD73 expression in macrophages. (A)** Heatmap of cytokine array results from LLCs one day (IR) or three days (Se) after 10 Gy-radiation. Data presented as mean of 3 biological replicates. **(B)** Relative mRNA expression of *P65* in LLCs harboring control (shNC) or p65 shRNAs (shp65). The indicated results represent the mean ± SEM of 4 independent experiments. **(C)** Heatmap of cytokine array results from LLCs harboring control (shNC) or p65 shRNAs (shp65) and treated as in **(A)**. Data presented as mean of 3 biological replicates. (**D-H**) The content of IL-6 **(D)**, IL-1β **(E)**, CXCL10 **(F)**, TNFα **(G)** and CCL5 **(H)** in the supernatant of LLCs in the corresponding groups detected by ELISA. **(I)** Relative mRNA expression of *Cd73* in BMDMs after co-culture with LLCs as described in **(C)** for 24 h. The indicated results represent the mean ± SEM of 3 independent experiments. **(J)** Flow cytometric analysis of CD73 expression on TAMs in LLCs harboring control (shNC) or p65 shRNAs (shp65) subcutaneous tumor and treated with radiotherapy (RT) (n = 6 per group). The indicated results represent the mean ± SEM, analyzed by one-way ANOVA. **(K)** Immunofluorescence staining of CD73 expression (green) on the surface of F4/80^+^ TAMs (red) in the tumor samples described in (E). Scale bar: 10 μm. *p < 0.05; **p < 0.01; ***p < 0.001; ns, not statistically significant.

There is growing evidence that SASP secreted by senescent cells can transmit information to other cells in the TME in a paracrine manner, promoting malignant progression 4, 5. Therefore, we asked whether senescent tumor cells induce macrophage CD73 expression in a SASP factors-dependent manner. Indeed, compared to control or 24 h post 10 Gy-irradiation, LLC cells on the third day after 10 Gy-irradiation showed an increased expression of various pro-inflammatory factors and chemokines, including IL-6, TNF-a, CCL5, CXCL10 **(Figure 2A)**. To validate the direct effect of SASP on macrophages-expressed CD73, LLC cells were transduced with short hairpin RNAs targeting the p65 subunit of NF-κB (shp65)** (Figure 2B, Figure S4A)**, an important transcription factor inducing SASP production but not required for senescence-mediated growth arrest. Inhibition of NF-κB in LLC cells reduced expression of diverse immunomodulatory and SASP factors on the third day after 10 Gy-irradiation, while the secretion of SASP cytokines in their culture supernatants had also been significantly diminished, including IL-6, IL-1β, CXCL10, TNFα and CCL5 **(Figure 2C-H)**. LLC cells with shp65 or shNC were then co-cultured with BMDMs after induction of senescence *in vitro*, and the upregulation of BMDM CD73 was partially rescued by p65 knockdown in senescent LLC cells** (Figure 2I)**. Further, we inoculated LLC subcutaneous tumors with shp65 and shNC in C57BL/6 mice *in vivo*, respectively. Consistently, inhibition of p65 significantly blocked the CD73 upregulation in macrophage in radiotherapy-induced senescence TME, and the proportion of CD73^+^F4/80^+^ cells in the TME was also reduced **(Figure 2J-K, Figure S4B)**. In addition, we used TPCA1, a potent NF-κB inhibitor, to inhibit SASP production in senescent LLC cells **(Figure S4C-H)**. Similarly, treatment of senescent LLC cells with TPCA1 did not cause substantial upregulation of CD73 in BMDMs **(Figure S4I)**. Thus, SASP factors secreted by senescent tumor cells are key factors influencing CD73 expression in macrophages.

### IL-6 secreted by senescent tumor cells up-regulates macrophages CD73 expression via JAK/STAT3 pathway

**Figure 3:**
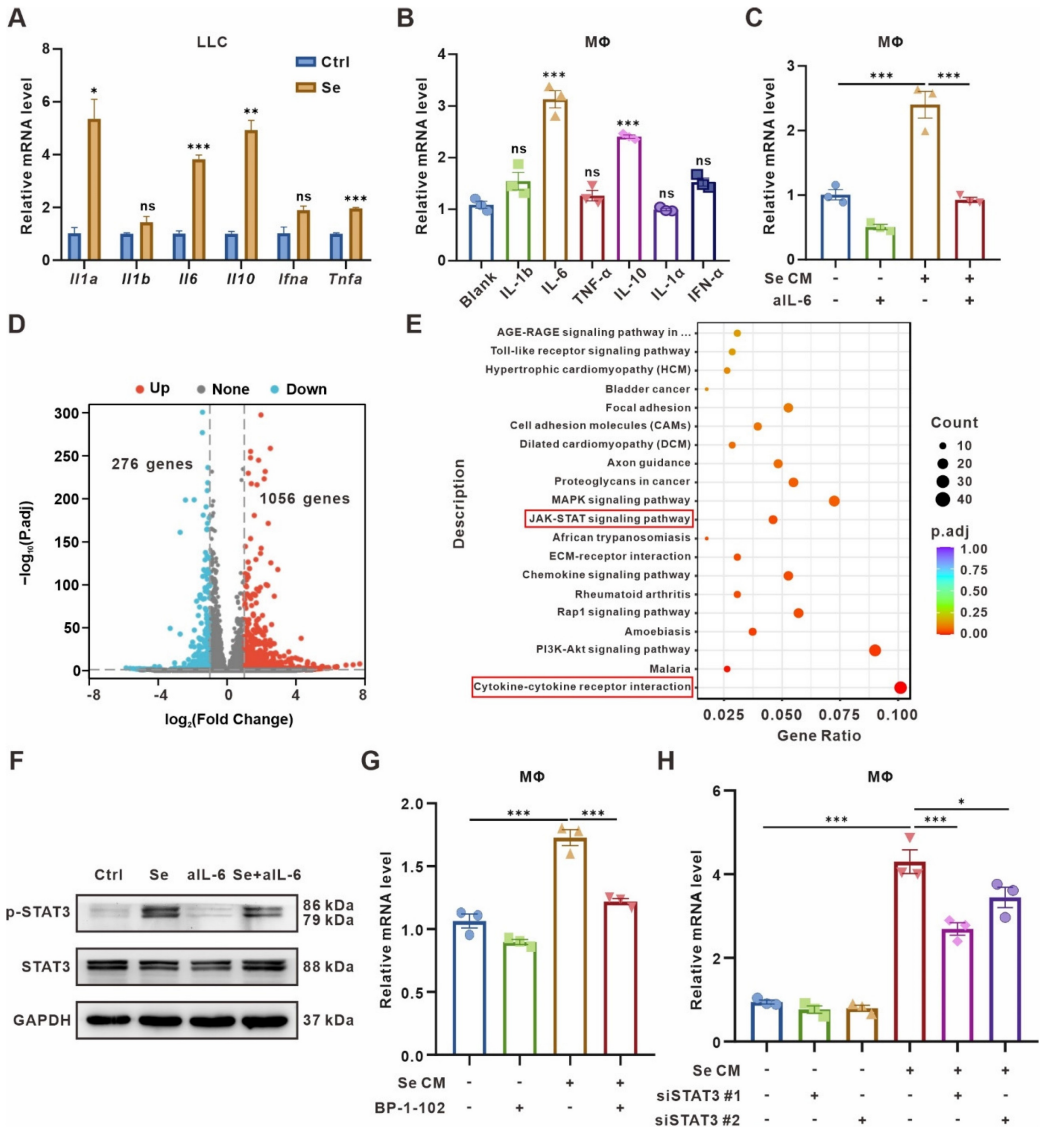
** CD73 expression on macrophages is induced by IL-6 and regulated by JAK/STAT3 pathway. (A)** Relative mRNA expression of *Il1a*, *Il1b*, *Il6*, *Il10*, *Ifna*, and *Tnfa* in senescent LLCs (Se). The indicated results represent the mean ± SEM of 3 independent experiments. **(B)** Relative mRNA expression of *Cd73* in BMDMs after treated with 20 ng/mL different cytokines for 24 h. The indicated results represent the mean ± SEM of 3 independent experiments. **(C)** Relative mRNA expression of *Cd73* in BMDMs after incubation with senescence-conditioned medium (Se CM) and/or 10 μg/mL IL-6-neutralizing antibodies (aIL-6) for 24 h. The indicated results represent the mean ± SEM of 3 independent experiments. **(D)** Volcano plot showing the upregulated, downregulated, or insignificantly differential expressed genes of BMDMs co-cultured with senescent or control LLCs for 24 h. **(E)** KEGG pathway enrichment revealing the activation of cytokine-cytokine receptor interaction and JAK/STAT signaling pathways as indicated in BMDMs co-cultured with senescent LLCs. **(F)** Representative Western blot for protein expression levels of pSTAT3, and STAT3 in BMDMs. **(G)** Relative mRNA expression of *Cd73* in BMDMs after treated with Se CM and/or BP-1-102 (5 μM) for 24 h. The indicated results represent the mean ± SEM of 3 independent experiments. **(H)** Relative mRNA expression of *Cd73* in BMDMs harboring STAT3 siRNAs (siSTAT3) and treated with Se CM for 24 h. The indicated results represent the mean ± SEM of 3 independent experiments. *p < 0.05; **p < 0.01; ***p < 0.001; ns, not statistically significant.

To seek out specific SASP factors promoting CD73 expression, we performed qPCR to test cytokines expression in senescent LLC cells, including *Il1α*, *Il1b*, *Il6*, *Il10*, *Ifnα*, and *Tnfα*. The expression level of *Il1α*, *Il6*, *Il10*, and *Tnfα* were significantly upregulated in senescent tumor cells **(Figure 3A)**. Further, we supplemented BMDMs *in vitro* with 20 ng/mL of different cytokines and measured the CD73 levels 24 h later. We found that IL-6 or IL-10 caused the CD73 upregulation in macrophages, with IL-6 causing the most remarkable changes **(Figure 3B)**. Meanwhile, ELISA assay also showed a significant elevation of IL-6 concentration in the culture supernatants of senescent LLC cells **(Figure S5A)**. Therefore, we added IL-6-neutralizing antibodies to Se CM and found the clearance of IL-6 reversed the CD73 upregulation in BMDMs **(Figure 3C)**.

To further determine the signaling pathways regulating CD73 elevation in BMDMs, RNA-Seq was performed on BMDMs co-culturing with senescent or control tumor cells for 24 h. BMDMs co-cultured with senescent tumor cells expressed 1056 genes up-regulated and 276 genes down-regulated compared with the control group **(Figure 3D, Table S6)**. Kyoto Encyclopedia of Genes and Genomes (KEGG) analyses showed significant enrichment of Cytokine-cytokine receptor interaction and JAK/STAT signaling pathways in BMDMs co-cultured with senescent tumor cells **(Figure 3E)**. As the IL-6/JAK/STAT3 signaling pathway has been implicated crucial in diverse malignancies 31, 32, we detected the expression of canonical markers in this pathway in BMDMs cultured with different CM. Phosphorylated STAT3 (pSTAT3) was upregulated in BMDMs treated with Se CM compared to CM from normal tumor cells (Ctrl), which could be rescued by IL-6-neutralized CM **(Figure 3F)**. To explore whether the CD73 upregulation in macrophages was driven by STAT3 in the Se CM, STAT3 inhibitor BP-1-102 was added to the Se CM. It suggested that inhibition of STAT3 reversed the CD73 upregulation in BMDMs **(Figure 3G)**. We also silenced STAT3 in BMDMs and found its rescued effect on Se CM induced CD73 upregulation **(Figure 3H, Figure S5B-C)**. In addition, we used the JAK1/2 inhibitor Ruxolitinib and demonstrated that inhibition of JAK1/2 partially reversed the CD73 elevation in BMDMs induced by Se CM **(Figure S5D)**. In conclusion, IL-6 secreted by senescent tumor cells facilitates macrophage CD73 expression via the JAK/STAT3 signaling pathway.

### Clearance of senescent cells confers antitumor effects but impedes T cell infiltration in TME

**Figure 4:**
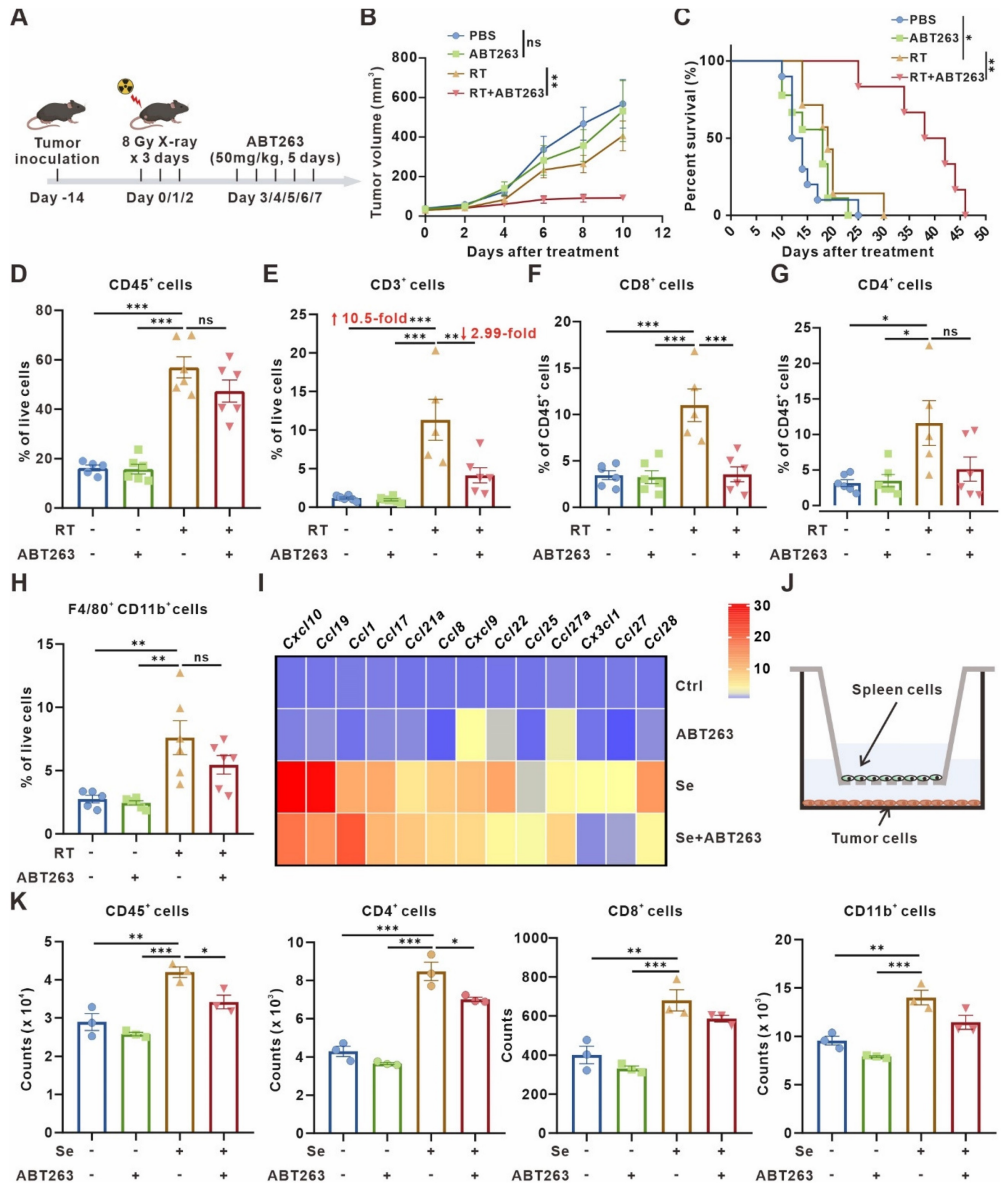
** Clearance of senescent cells inhibits tumor growth but disrupts T-cell infiltration. (A)** Schematic of experiment to assess efficacy of ABT263-senescence removal treatment in mouse model. **(B)** Tumor growth curves of LLC subcutaneous transplant model in corresponding treatment groups (n = 6 to 7 per group), analyzed by 2-way ANOVA. **(C)** Kaplan-Meier survival plot of LLC lung cancer-bearing mice in the corresponding treatment groups described in (B) (n = 6 to 10 per group). **(D-H)** Flow cytometry analysis of changes in the immune cells in the TME of LLC subcutaneous transplant model that underwent different treatments (n = 5 to 6 per group). **(I)** Heatmap of T cell-related chemokines results from senescent LLCs and/or treated with ABT263(100 nM) for 48 h. Data presented as mean of 3 biological replicates. **(J)** Pattern diagram of transwell migration assay. **(K)** ABT263 inhibited the chemotactic effects of senescent LLCs on CD45^+^ cells, CD4^+^ T cells, CD8^+^ T cells, and CD11b^+^ myeloid cells. The indicated results represent the mean ± SEM of 3 independent experiments, analyzed by one-way ANOVA. *p < 0.05; **p < 0.01; ***p < 0.001; ns, not statistically significant.

As mentioned above, senescent tumor cells upregulated CD73 in macrophages, which elicited accumulation of the immunosuppressive adenosine in TME. Therefore, we wondered whether the clearance of senescent cells in TME would be beneficial for the activation of antitumor immunity. Following the procedure shown in **Figure 4A**, senescent cells in murine subcutaneous LLC model were scavenged **(Figure 4A)**. Compared with radiotherapy or ABT263 treatment alone, the tumor growth was significantly inhibited and the mice survival was prolonged in the combination treatment group** (Figure 4B-C)**. To elucidate the alteration of tumor immune microenvironment after combination treatment, we detected the immune cell infiltration of murine LLC subcutaneous tumors by flow cytometry **(Figure S6A-B)** and found that compared with control or ABT263 treatment alone, radiotherapy alone induced a 10.5-fold and 11.67-fold increment in T cell infiltration respectively. The ratio of CD8^+^GrzB^+^/CD8^+^ T cells and CD4^+^GrzB^+^/CD4^+^ T cells were significantly increased **(Figure 4D-G, Figure S6C-F)**. Surprisingly, the proportion of T cells in the TME was reduced to one-third after removing senescent cells in the combined treatment group compared with the radiotherapy alone, especially for CD8^+^ T cells **(Figure 4E-F)**. Although the alteration on the proportion of CD8^+^GrzB^+^ T cells and CD4^+^GrzB^+^ T cells was not apparent after scavenging senescent cells, the absolute numbers were still significantly reduced due to the decreased total number of chemotactic CD4^+^ and CD8^+^ cells in the microenvironment **(Figure S6C-F)**. These results indicated that the removal of senescent cells suppressed T cells recruitment, but did not alter the function of T cells. In addition, the combination treatment had no significant impact on the recruitment and polarization of CD11b^+^F4/80^+^ macrophages compared with radiotherapy alone **(Figure 4H, Figure S6G-H)**.

It was reported that treatment-induced senescent tumor cells secrete SASP-associated cytokines to promote T cell infiltration 33, so we wondered whether the reduction of T cells induced by radiotherapy combined with ABT263 treatment is related to the chemotaxis of senescent cells to T cells. We measured the expression of various T cell chemotaxis-related cytokines, including CXCL9, CXCL10, CCL19, and CCL8, in 10 Gy-irradiation-induced senescent LLC cells treated with or without ABT263 *in vitro* by qPCR. We found diminished expression of T cell-related chemokines after clearing senescent cells **(Figure 4I)**. Further, using co-culture system with tumor cells in the lower chamber and mouse spleen single cell suspensions in the upper chamber, flow cytometry was utilized to detect the number of different types of immune cells converging to the lower chamber **(Figure 4J)**. It indicated that senescent tumor cells exhibited remarkable chemotaxis to CD45^+^ cells, CD4^+^ T cells, CD8^+^ T cells, and CD11b^+^ myeloid cells. Nevertheless, the combination of ABT263 resulted in a significant reduction of CD45^+^ cells and CD4^+^ T cells chemotactic to the lower chamber **(Figure 4K)**. Collectively, these results demonstrate that removal of senescent cells from TME suppresses tumor growth, but also hinders T cells infiltration.

### Targeted inhibition of CD73 in the aging microenvironment suppresses tumors while triggering anti-tumor immunity

**Figure 5:**
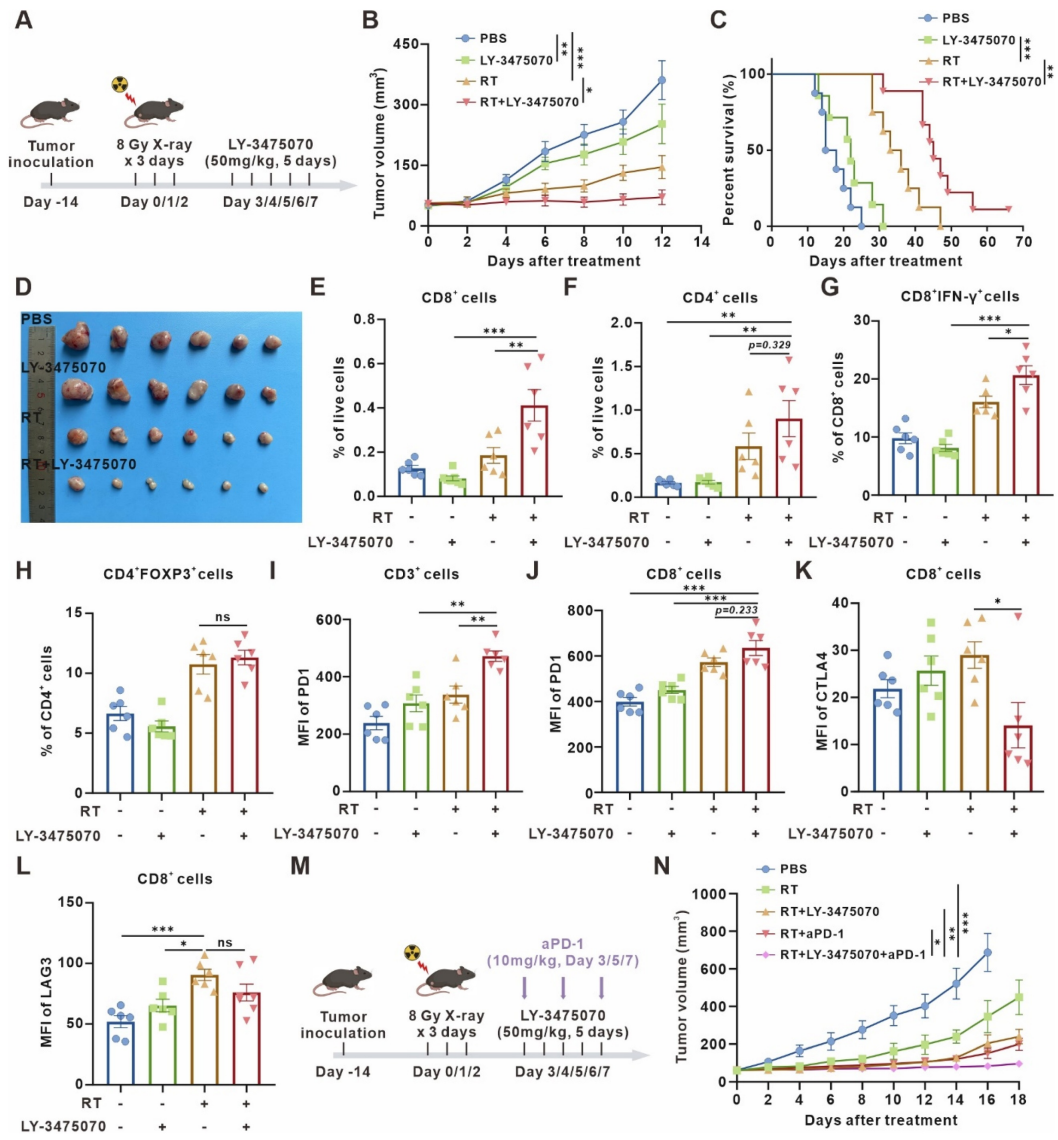
** Targeted inhibition of CD73 in the senescent TME inhibits tumor growth and triggers anti-tumor immunity. (A)** Schematic of experiment to assess efficacy of LY-3475070 in the senescent TME in mouse model. **(B)** Tumor growth curves of LLC subcutaneous transplant model in corresponding treatment groups (n = 6 to 7 per group), analyzed by 2-way ANOVA. **(C)** Kaplan-Meier survival plot of LLC lung cancer-bearing mice in the corresponding treatment groups described in (B) (n = 7 to 9 per group). **(D)** Photo of dissected tumors in each group. **(E-H)** Flow cytometry analysis of changes in the immune cells in the TME of LLC subcutaneous transplant model that underwent different treatments (n = 6 per group). **(I-J)** Flow cytometry analysis of PD-1 expression on the surface of CD3^+^ T cells, and CD8^+^ T cells of LLC subcutaneous transplant model that underwent different treatments (n = 6 per group). **(K-L)** Flow cytometry analysis of CTLA4 **(K)** and LAG3 **(L)** expression on the surface of CD8^+^ T cells of LLC subcutaneous transplant model that underwent different treatments (n = 6 per group). **(M)** Schematic of experiment to assess efficacy of LY-3475070 combined with anti-PD-1 mAb in the senescent TME in mouse model. **(N)** Tumor growth curves of LLC subcutaneous transplant model in corresponding treatment groups (n = 6 to 7 per group), analyzed by 2-way ANOVA. *p < 0.05; **p < 0.01; ***p < 0.001; ns, not statistically significant.

Since the removal of senescent cells brings detrimental effects on antitumor immunity, we decided to retain senescent cells in the microenvironment, while preserving the immune-activating effects of senescence. Thus, we turn to explore the potential of CD73 as an antitumor therapeutic target in the senescent microenvironment. Consistent with our speculation, radiotherapy combined with CD73 inhibitor LY-3475070 induced apparent tumor growth delay and prolonged survival compared with radiotherapy alone, and subcutaneous tumors disappeared completely during treatment in one of nine mice in the combined treatment group **(Figure 5A-D)**. The results were re-validated in a cisplatin chemotherapy-induced senescence model. Consistently, cisplatin combined with LY-3475070 significantly suppressed tumor growth and prolonged mice survival compared with cisplatin treatment alone. Likewise, one of seven mice in the combined treatment group achieved complete cure **(Figure S7)**. The immune infiltration of subcutaneous tumors in different groups was examined, and it was noticed that the combination therapy further increased T cells, escpecially the proportion of CD3^+^CD8^+^IFN-γ^+^ T cells compared with radiotherapy alone **(Figure 5E-G)**. We observed no significant difference in the frequency of Foxp3^+^ T regulatory cells between groups **(Figure 5H)**.

Furthermore, we examined the expression of T cell surface exhaustive markers, such as PD-1, CTLA4, and LAG3, figuring out PD-1 upregulation **(Figure 5I-J)** and CTLA4 downregulation **(Figure 5K)** caused by combined therapy. However, there was no significant difference in LAG3 expression **(Figure 5L)**. Then, a further combination of anti-PD-1 monoclonal antibody (mAb) was performed. The combined therapy resulted in remarkable tumor growth inhibition compared with radiotherapy combined with LY-3475070 or anti-PD-1 mAb **(Figure 5M-N)**. These results illustrated that targeting CD73 in the senescent TME exerted remarkable anti-tumor effects, and effectively improved the immunotherapeutic efficacy of anti-PD-1 mAb.

### Validation of the clinical relevance between cellular senescence and CD73^+^ macrophages in lung adenocarcinoma

**Figure 6:**
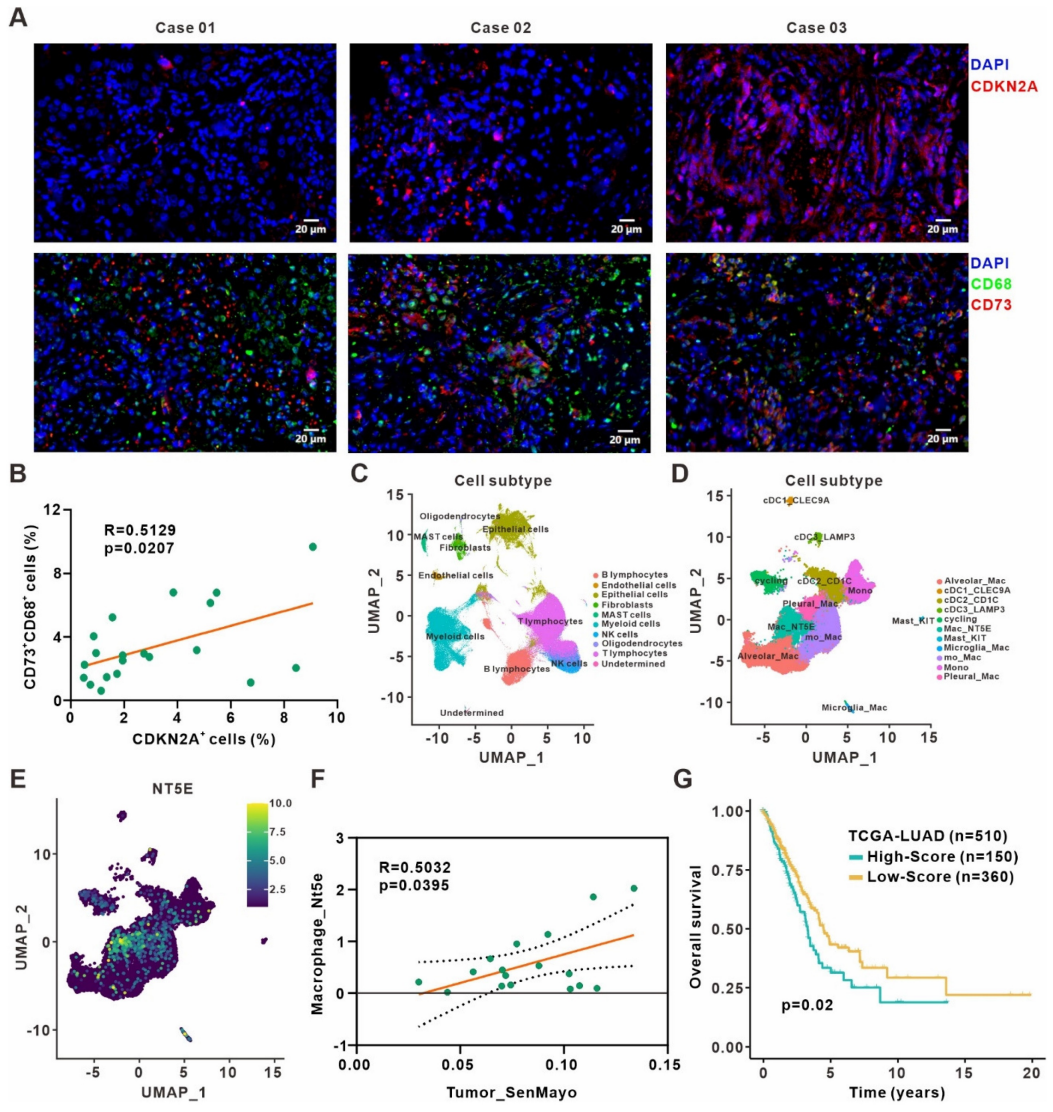
** Clinical relevance between senescence and CD73^+^ macrophages in lung adenocarcinoma. (A)** Representative immunofluorescence staining of senescence-associated marker CDKN2A (red) and CD73 expression (red) on the surface of macrophages (green) in the surgically resected specimens from primary lung adenocarcinoma patients. Scale bar: 20 μm. **(B)** Linear regression analysis between the frequencies of CDKN2A^+^ senescent cells and CD73^+^CD68^+^ macrophages in specimen sections as described in **(A)**. **(C)** UMAP plot of single cells colored by the major cell lineages. **(D)** UMAP plot of myeloid cells, color-coded by clusters and cell subsets as indicated. **(E)** UMAP plot of NT5E expression in myeloid cells as described in **(D)**. **(F)** Linear regression analysis between the senescence gene set (SenMayo) expression of tumor cells and the CD73 expression of macrophages in the single-cell dataset. **(G)** Survival based on the expression level of senescent macrophage gene set in the TCGA lung adenocarcinoma cohort (log-rank test).

We validated our findings in surgically resected specimens from 20 primary lung adenocarcinoma patients by immunofluorescence staining and revealed a positive correlation between frequencies of CDKN2A^+^ senescent cells and CD73^+^CD68^+^ macrophages **(Figure 6A-B)**. Besides, the analysis of The Cancer Genome Atlas (TCGA) lung adenocarcinoma dataset also indicated that CDKN1A expression was positively associated with immune score and cytotoxic cells enrichment. The expression of CDKN1A and CD73 also showed a positive correlation **(Figure S8A-C)**. Moreover, we reanalyzed the published single-cell transcriptome profiling of metastatic lung adenocarcinoma 28 to divide all cells from different samples into ten clusters **(Figure 6C, Figure S8D)**, and further sub-clustering of myeloid cells revealed eleven clusters **(Figure 6D, Figure S8E)**. Analysis of CD73 expression in different myeloid cell clusters showed a high expression in macrophages **(Figure 6E)**. We also quantified the senescence gene set (SenMayo) of tumor cells, the SASP gene set (R.HSA.2559582) 34 of tumor cells, and CD73 expression of macrophages in the single-cell dataset, respectively. The analysis revealed positive correlation between senescence gene set of tumor cells and macrophages CD73 expression **(Figure 6F)**, while SASP expression in tumor cells and CD73 in macrophages were also positively correlated **(Figure S8F)**. Finally, to investigate the prognostic value of remodeled TAMs in the senescence microenvironment, top 102 differentially expressed genes from our macrophage RNA-Seq were defined as the senescent macrophage gene set **(Table S7)**. Patients in the TCGA lung adenocarcinoma cohort were classified into high and low-score groups according to the expression level of this gene set. Patients in the high-score group had shorter survival and poorer prognosis **(Figure 6G)**. It suggested that macrophages remodeled by the senescent microenvironment probably exerted a pro-tumor role, and early detection of the senescent macrophage gene set might have certain prognostic significance for patients.

## Discussion

**Figure 7:**
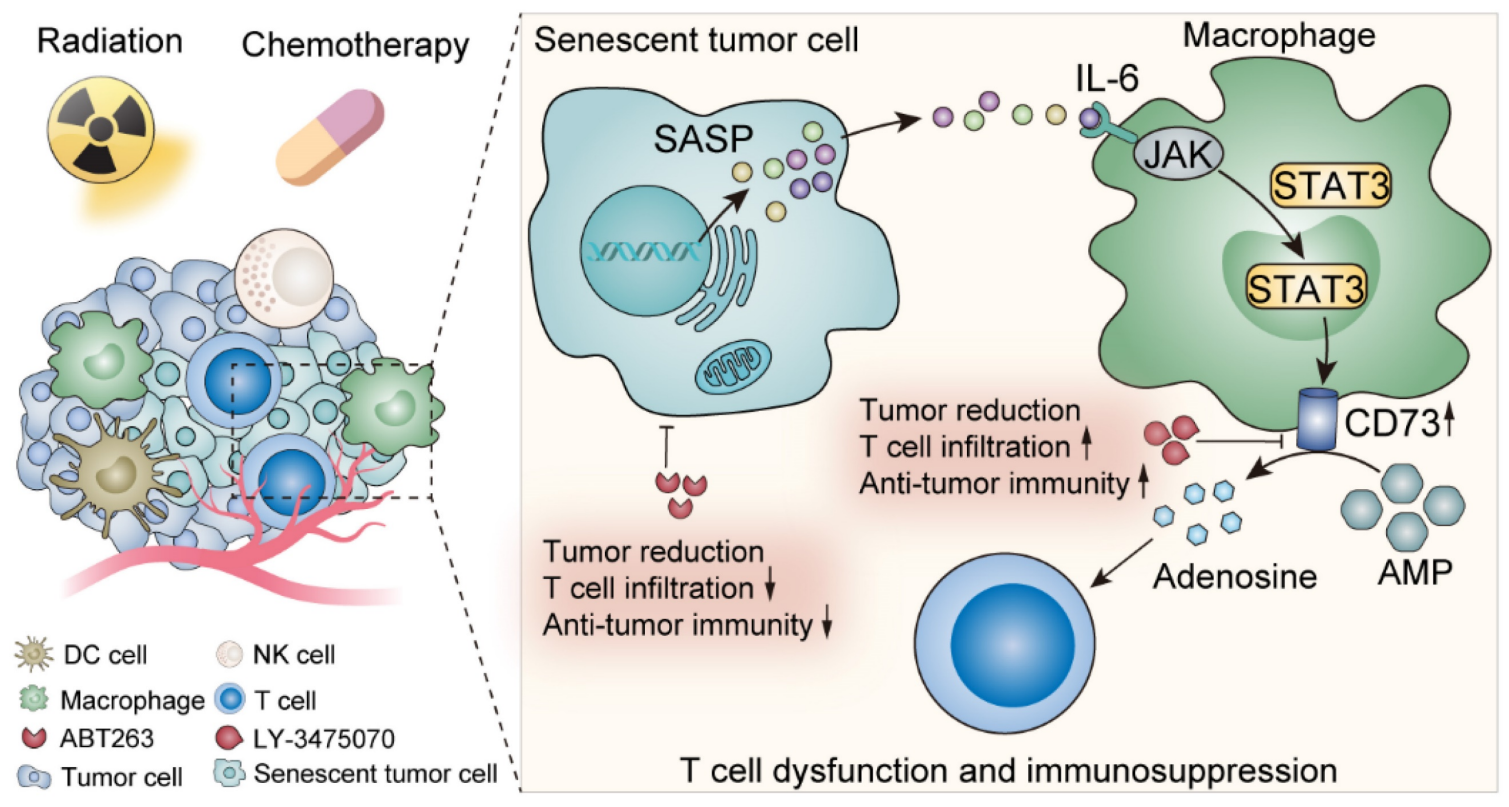
Schematic illustration shows the effects of senescent tumor cells on macrophages and potential therapeutic targets.

Here we elucidated the metabolic features of senescent TME and discovered a prominent elevation of adenosine, in which TAMs made an appreciable contribution. IL-6 secreted by senescent tumor cells acted on TAMs, up-regulated TAMs CD73 expression through JAK/STAT3 signaling pathway, and suppressed anti-tumor immunity. In contrast, CD73 inhibitors greatly motivated anti-tumor immunity under senescent conditions, which provided perspectives on potent anti-tumor targets exploration **(Figure 7)**.

Cellular senescence has long been generally recognized as a protective mechanism against oncogenesis 35-37. Nevertheless, accumulating evidence reveals the exact opposite phenomenon: senescent cells spur tumor growth and promote malignancy in some cases 37, 38. Removal of senescent cells, characteristically expressing the cell cycle inhibitor p16^-INK4a^, from aging mice not only delayed age-related symptoms, but also diminished the incidence of both spontaneous tumors and cancer-related death 39. However, based on the complexity of senescence, indiscriminately removing senescent cells does not achieve the desirable anti-tumor effect. Seeking more precise targets is essential to achieve more satisfying curative effect against cancer. We evidenced that CD73 upregulation in TAMs induced by senescent tumor cells suppressed anti-tumor immunity, while inhibition of CD73 showed the opposite effect, suggesting that CD73 is a critical brake of anti-tumor immunity in the aging microenvironment.

With the successful clinical application of immune-checkpoint inhibitors (ICIs) in recent years, immunotherapy has become an attractive option for tumor treatment 40. However, most patients still fail to benefit from it, the objective response rates (ORRs) of PD-1/PD-L1 monotherapy are only about 20% 41. Combination therapy is expected to improve its efficacy, and the combined drugs or radiation cause senescence of tumor cells and stromal cells in TME 42, 43. It has been demonstrated that senescent tumor cells facilitate T-cell infiltration into the TME, turning the “cold tumor” into “hot tumor” 33. Moreover, senescent cells are powerfully immunogenic and effectively trigger anti-tumor immune responses mediated by CD8^+^ T cells 44, which is likely to be an important factor to improve immunotherapy response by combination therapy. We showed that senescent cells do recruit immune cells but also suppress anti-tumor immunity by inducing TAMs to express CD73. CD73 inhibitors further enhances the effect of combination therapy, which provides new initiatives to improve the efficacy of immunotherapy.

Currently, metabolism and TME are the focus of intense research. In nutritional deficiency, hypoxia, and acidic microenvironment, tumor cells are metabolically reprogrammed to enable their growth, proliferation, and survival 45. Therefore, targeting distinct metabolic pathways or metabolites has become a novel idea for probing anti-tumor therapies. However, how senescent tumor cells influence microenvironmental metabolism has not been elucidated yet, so in this study we investigated and complemented the metabolic features of the senescent TME by metabonomic analysis on the TIF after radiotherapy and radiotherapy combined with ABT263 to clear senescence, avoiding the effects of radiotherapy itself on the TME. We found that the immunosuppressive molecule adenosine in the senescent microenvironment was significantly elevated, suggesting that it may be a probable mechanism by which senescence acts as a negative regulator, and provides an alternative idea for further precise targeting senescent microenvironment to improve antitumor efficacy from a metabolic perspective.

Tumor-associated macrophages are important innate immune cells with complexity in the TME. We found that CD73 was predominantly expressed in M1-type macrophages in the senescent microenvironment and a positive correlation was shown between the expression of M1 marker CD86 and CD73. This seems to be contrary to the perception that M1-type TAMs promote anti-tumor immunity 48, however this is not the case. Previous studies have also revealed that pro-inflammatory factors such as IFN-γ and LPS, which induce macrophages to M1 polarization, also promote the upregulation of PD-L1 in TAMs, thereby inhibiting T cell activation and function 49-52. This is also indicative of the admixture of macrophages rather than a simple division into absolute M1 and M2 types. In addition, we identified that senescent tumor cells up-regulate macrophage CD73 expression via the SASP-IL-6-JAK/STAT3 signaling pathway, and validated this in lung cancer clinical specimens and databases. Finally, analysis of macrophage RNA-Seq results led to the construction of a senescent macrophage gene set, the high expression of which in lung cancer suggests a poorer prognosis, which also lays the foundation for improving anti-tumor efficacy by targeting macrophages in the senescent environment in the future.

Extracellular adenosine production is a critical immunosuppressive regulatory pathway in TME, which is generated mainly through the cascade hydrolysis reaction of CD39 and CD73, and the latter is the rate-limiting enzyme 25. CD73 is widely expressed in a variety of cells in the TME, including tumor cells, endothelial cells, stromal cells, and infiltrating immune cells 53, 54. It is reported that CD73^+^ B cells are capable of producing adenosine to regulate the immune responses of CD4^+^ and CD8^+^ T cells 55. In addition, CD73-expressing Treg cells are also essential for maintaining tumor growth 56, 57. In our study, CD73 expression on TAMs was pivotal in the senescent microenvironment and was the main contributor to adenosine accumulation. This also provides subsequent insights into specifically targeting macrophage CD73 expression in the senescent background.

In conclusion, our findings first evidenced the adenosine accumulation in the senescent TME and revealed the crosstalk among senescent tumor cells, TAMs, and adenosine in TME. Senescent tumor cells derived IL-6 induced the upregulation of TAMs CD73 expression through the JAK/STAT3 signaling pathway. From a therapeutic perspective, directly targeting CD73, rather than clearing senescent cells, activates anti-tumor immunity while inhibiting tumor growth. It suggests a promising future for the application of CD73 inhibitors or monoclonal antibodies.

## Conclusions

In summary, we identified metabolic profiles of the senescent tumor microenvironment, highlighted by elevated adenosine. The elevated CD73 expression in TAMs contributed predominantly to adenosine accumulation. Mechanistically, senescent tumor cells derived IL-6 induces the upregulation of TAMs CD73 expression through JAK/STAT3 signaling pathway. Targeting CD73, rather than senescent cell removal, activates anti-tumor immunity and enhances the efficacy of anti-PD-1. It provides insights into the exploration towards more precise and efficient anti-tumor strategy in the senescent microenvironment.

## Supplementary Material

Supplementary figures and tables.

## Abbreviations

ANOVA: analysis of variance
ATCC: American Tissue Culture Collection
BMDCs: bone marrow-derived dendritic cells
BMDMs: bone marrow-derived macrophages
CFSE: Carboxyfluorescein succinimidyl ester
Clo: Clodronate liposomes-Anionic
CM: Conditioned medium
DMEM: Dulbecco's Modified Eagle's Medium
FBS: Fetal Bovine Serum
GrzB: Granzyme B
ICIs: immune-checkpoint inhibitors
IL-6: Interleukin 6
KEGG: Kyoto Encyclopedia of Genes and Genomes
LC-MS/MS: liquid chromatography-tandem mass spectrometry
LLC: Lewis lung cancer cells
mAb: monoclonal antibody
ORRs: objective response rates
PCs: principal components
PMA: Phorbol 12-myristate 13-acetate
pSTAT3: phosphorylated STAT3
SA-β-gal: senescence-associated β-galactosidase
SASP: senescence-associated secretory phenotypes
scRNA-seq: Single-cell RNA sequencing
Se CM: Senescence-conditioned medium
TAMs: tumor-associated macrophages
TCGA: The Cancer Genome Atlas
TIF: tumor interstitial fluid
TLDs: thermo-luminescent dosimeters
TME: tumor microenvironment
TPM: transcripts per million
UMIs: unique molecular identifiers
VIP: variable importance in projection
