# Supplementary material for: Tumor cell senescence-induced macrophage CD73 expression is a critical metabolic immune checkpoint in the aging tumor microenvironment
Source: Theranostics. 2024 Jan 20;14(3):1224–40. doi: 10.7150/thno.91119 (PMC10845200; doi:10.7150/thno.91119)
Supplement: Supplementary file 1 — Supplementary figures and tables. [file thnov14p1224s1.zip › Supplementary materials.pdf]

Supplementary materials

Tumor cell senescence-induced macrophage CD73 expression is a critical metabolic immune checkpoint in the aging tumor microenvironment

Yue Deng[1,2,3†], Qinyan Chen[1,2,3†], Xiao Yang[1,2,3†], Yajie Sun[1,2,3], Bin Zhang[1,2,3], Wenwen Wei[1,2,3], Suke Deng[1,2,3], Jingshu Meng[1,2,3], Yan Hu[1,2,3], Yijun Wang[1,2,3], Zhanjie Zhang[1,2,3], Lu Wen[1,2,3], Fang Huang[1,2,3], Chao Wan[1,2,3*], Kunyu Yang[1,2,3*]

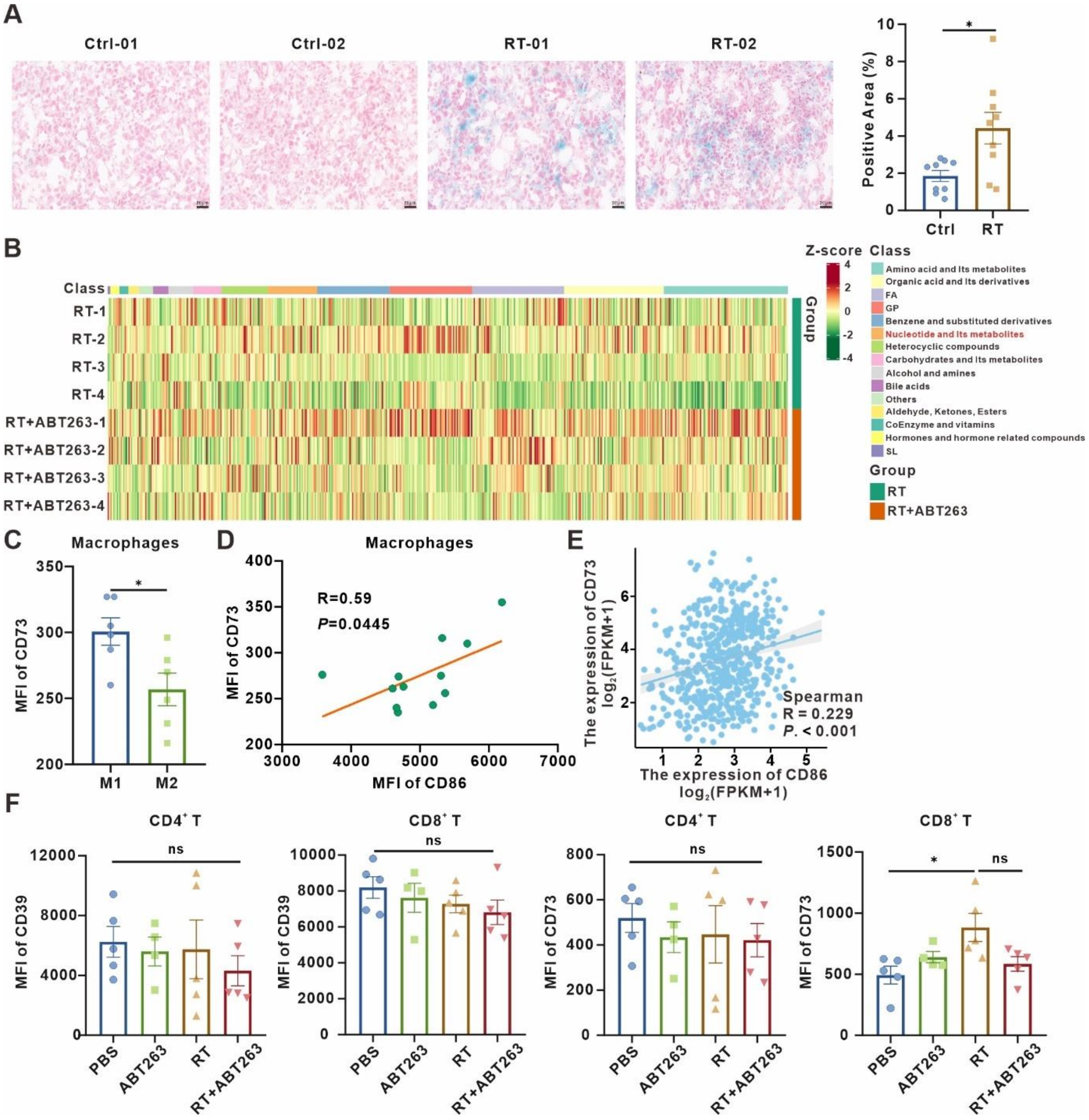

Figure S1. Characterization of the senescent TME in vivo.

(A) LLC murine subcutaneous tumors senescence detected by SA-β-Gal staining assay and the statistics of staining positive area. Scale bar: 20 μm. Mean ± SEM (3 microscopic fields at 400× magnification per mice to evaluate). (B) Heatmap showing the differential metabolites between the senescence (RT) and senescence-clearing (RT+ABT263) TIF detected by LC-MS/MS. (C) Flow cytometric analysis of CD73 expression on M1- and M2-type macrophages in LLC subcutaneous tumor under senescent TME (n = 6). (D) Linear regression analysis between the MFI of CD86 and CD73 in macrophages in the TME (n = 12). (E) Association between the expression of CD86 and CD73 in TCGA lung adenocarcinoma dataset. (F) Flow cytometric analysis of CD39 and CD73 expression on $CD4^+$ T cells and $CD8^+$ T cells in LLC

subcutaneous tumor under various treatment conditions (n = 4 to 5 per group), analyzed by one-way ANOVA. *p < 0.05; ns, not statistically significant.

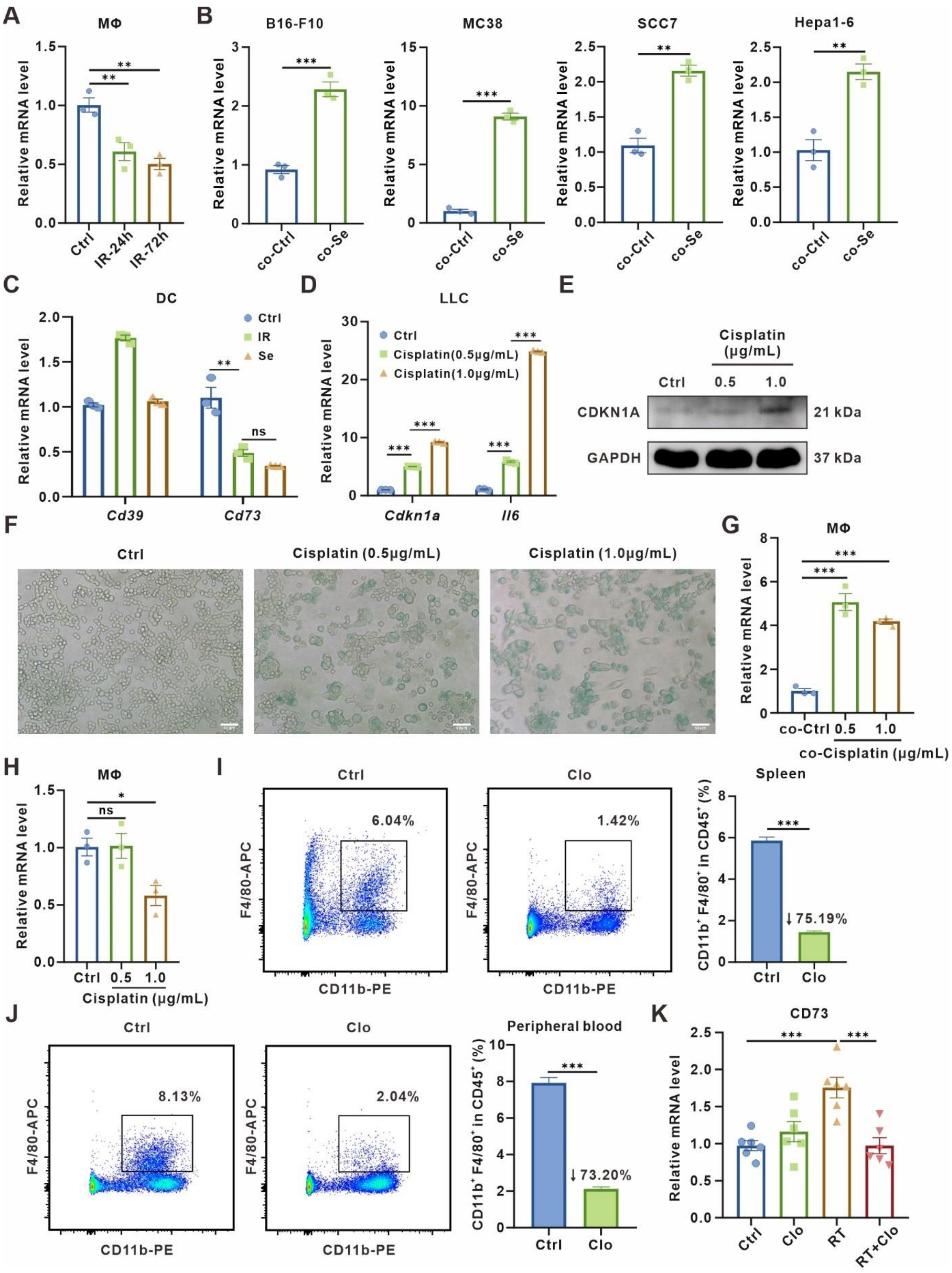

Figure S2. Senescent tumor cells cause upregulation of CD73 expression in macrophages.

(A) Relative mRNA expression of *Cd73* in BMDMs 24 h and 72 h after 10 Gy-radiation (IR). The indicated results represent the mean ± SEM of 3 independent experiments. (B) Relative mRNA expression

of *Cd73* in BMDMs after co-culture with various senescent tumor cells (B16-F10, MC38, SCC7, and Hepa1-6). The indicated results represent the mean ± SEM of 3 independent experiments. (C) Relative mRNA expression of *Cd39* and *Cd73* in BMDCs after co-culture with Ctrl LLCs (Ctrl), LLCs one day after 10 Gy-radiation (IR), or LLCs three days after 10 Gy-radiation (Se) for 24 h. The indicated results represent the mean ± SEM of 3 independent experiments. (D) Relative mRNA expression of senescence-associated genes in LLCs three days after pretreated with 0.5 μg/mL or 1.0 μg/mL cisplatin for 24 h. The indicated results represent the mean ± SEM of 3 independent experiments. (E) Western blot identifying the expression changes of CDKN1A in LLCs described in (D). (F) LLCs described in (D) senescence detected by SA-β-Gal staining assay. Scale bar: 50 μm. (G) Relative mRNA expression of *Cd73* in BMDMs after co-culture with LLCs as described in (D). The indicated results represent the mean ± SEM of 3 independent experiments. (H) Relative mRNA expression of *Cd73* in BMDMs after treated with 0.5 μg/mL or 1.0 μg/mL cisplatin. The indicated results represent the mean ± SEM of 3 independent experiments. (I) Macrophage clearance efficiency in mouse spleen detected by flow cytometry (n = 3 per group). The indicated results represent the mean ± SEM. (J) Macrophage clearance efficiency in mouse peripheral blood detected by flow cytometry (n = 3 per group). The indicated results represent the mean ± SEM. (K) Relative mRNA expression of *Cd73* in LLC murine subcutaneous tumors in the corresponding treatment groups (n = 6 per group). The indicated results represent the mean ± SEM. *$p < 0.05$; **$p < 0.01$; ***$p < 0.001$; ns, not statistically significant.

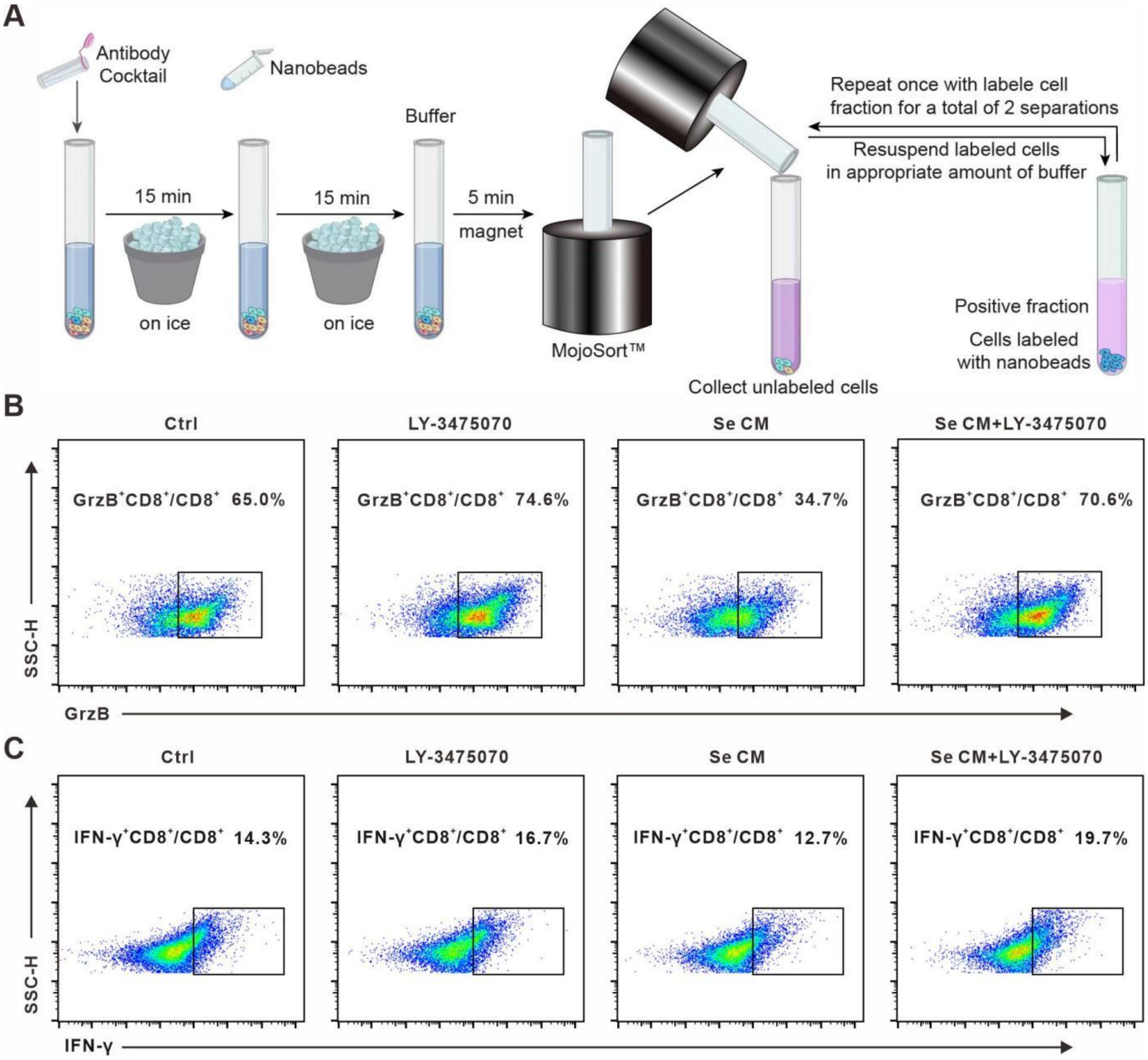

Figure S3. Macrophages co-cultured with senescent tumor cells inhibits the function of T cells.

(A) Schematic diagram of the $CD8^+$ T cells sorting process. (B-C) Representative flow cytometry dot plots of the percentage of $GrzB^+CD8^+$ T cells in $CD8^+$ T cells (B) and the percentage of IFN-γ^+CD8^+ T cells in $CD8^+$ T cells (C) in the corresponding group.

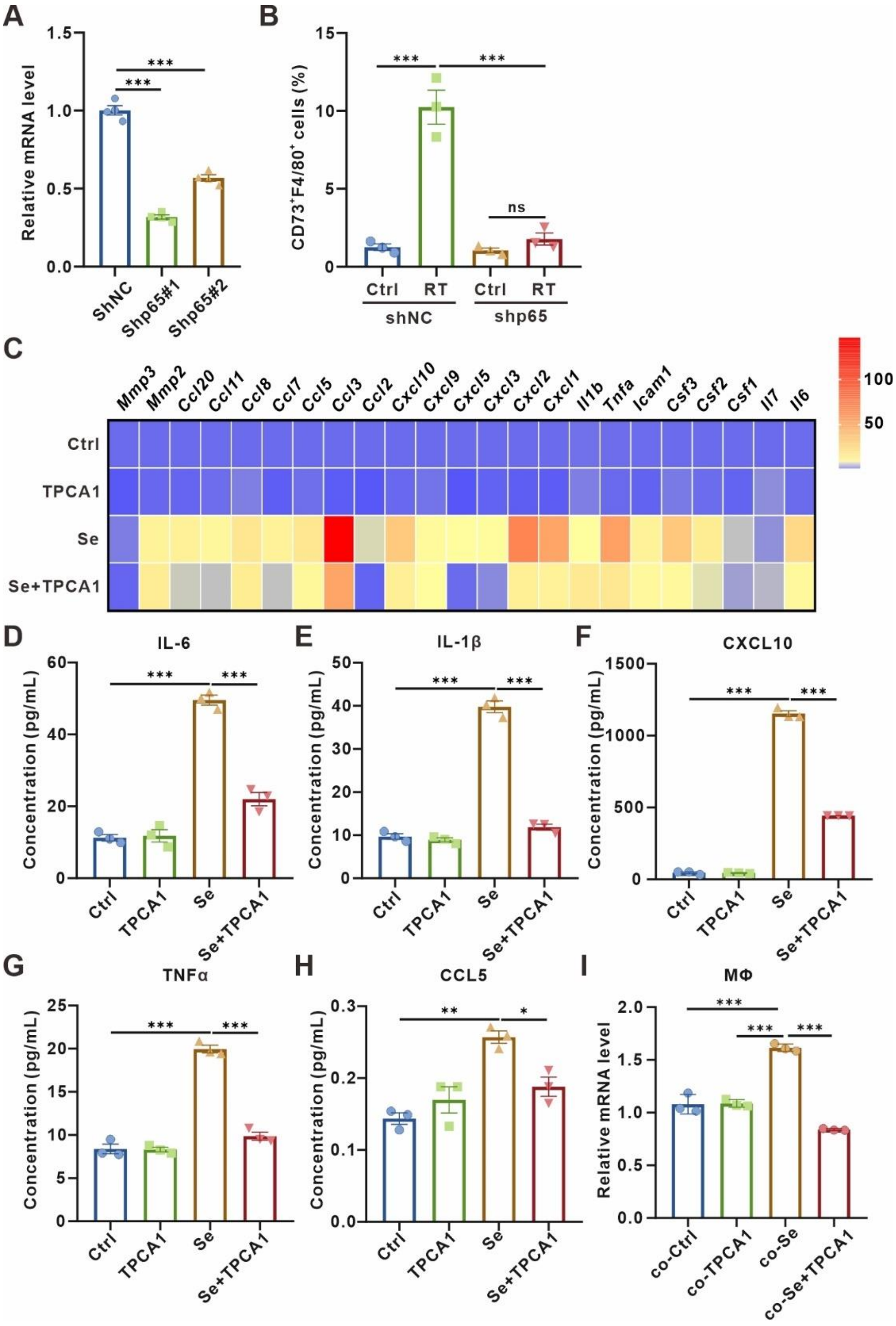

Figure S4. TPCA1 inhibits the upregulation of BMDMs CD73 by senescent LLCs.

(A) Relative mRNA expression of *P65* in LLCs harboring control (shNC) or p65 shRNAs (shp65). The indicated results represent the mean ± SEM of 4 independent experiments. (B) The proportion of CD73^{+}F4/80^{+} cells in LLCs shNC or shp65 subcutaneous tumor sections. (C) Heatmap of cytokine array results from senescent LLCs and/or treated with TPCA1 (1 μM) for 24 h. Data presented as mean of 3 biological replicates. (D-H) The content of IL-6 (D), IL-1β (E), CXCL10 (F), TNFα (G) and CCL5 (H) in

the supernatant of LLCs in the corresponding groups detected by ELISA. (I) Relative mRNA expression of *Cd73* in BMDMs after co-culture with senescent LLCs which were pretreated with TPCA1 (1 μM) for 24 h. The indicated results represent the mean ± SEM of 3 independent experiments. *$p < 0.05$; **$p < 0.01$; ***$p < 0.001$; ns, not statistically significant.

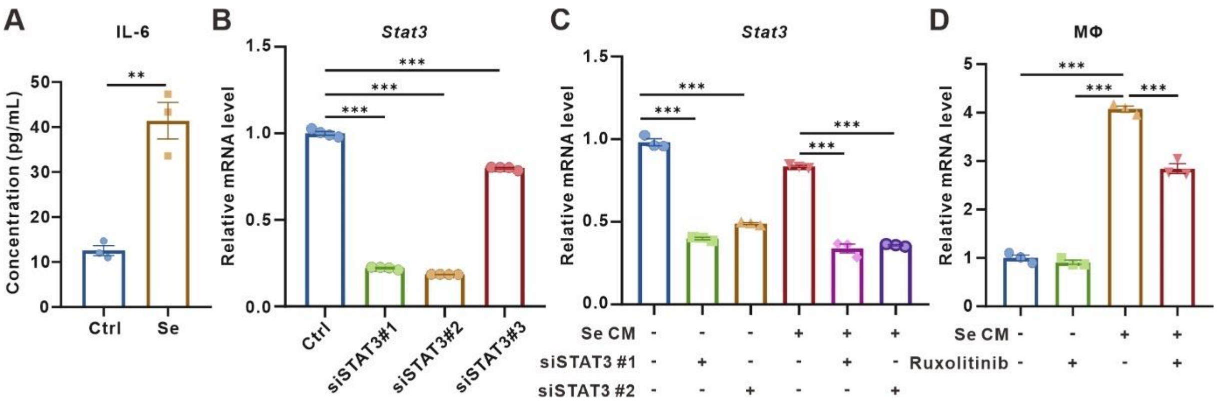

Figure S5. Upregulation of macrophage CD73 is mediated by JAK/STAT3 pathway.

(A) IL-6 content in the supernatant of senescent LLCs detected by ELISA. (B) RT-qPCR analysis to verify the expression levels of *Stat3* mRNAs in BMDMs harboring control (Ctrl) or STAT3 siRNAs (siSTAT3). The indicated results represent the mean ± SEM of 4 independent experiments. (C) RT-qPCR analysis to verify the mRNAs expression of *Stat3* in BMDMs in the corresponding groups. The indicated results represent the mean ± SEM of 3 independent experiments. (D) Relative mRNA expression of *Cd73* in BMDMs after co-culture with Se CM and treated with Ruxolitinib (5 μM) for 24 h. The indicated results represent the mean ± SEM of 3 independent experiments. $^{**}p < 0.01$; $^{***}p < 0.001$.

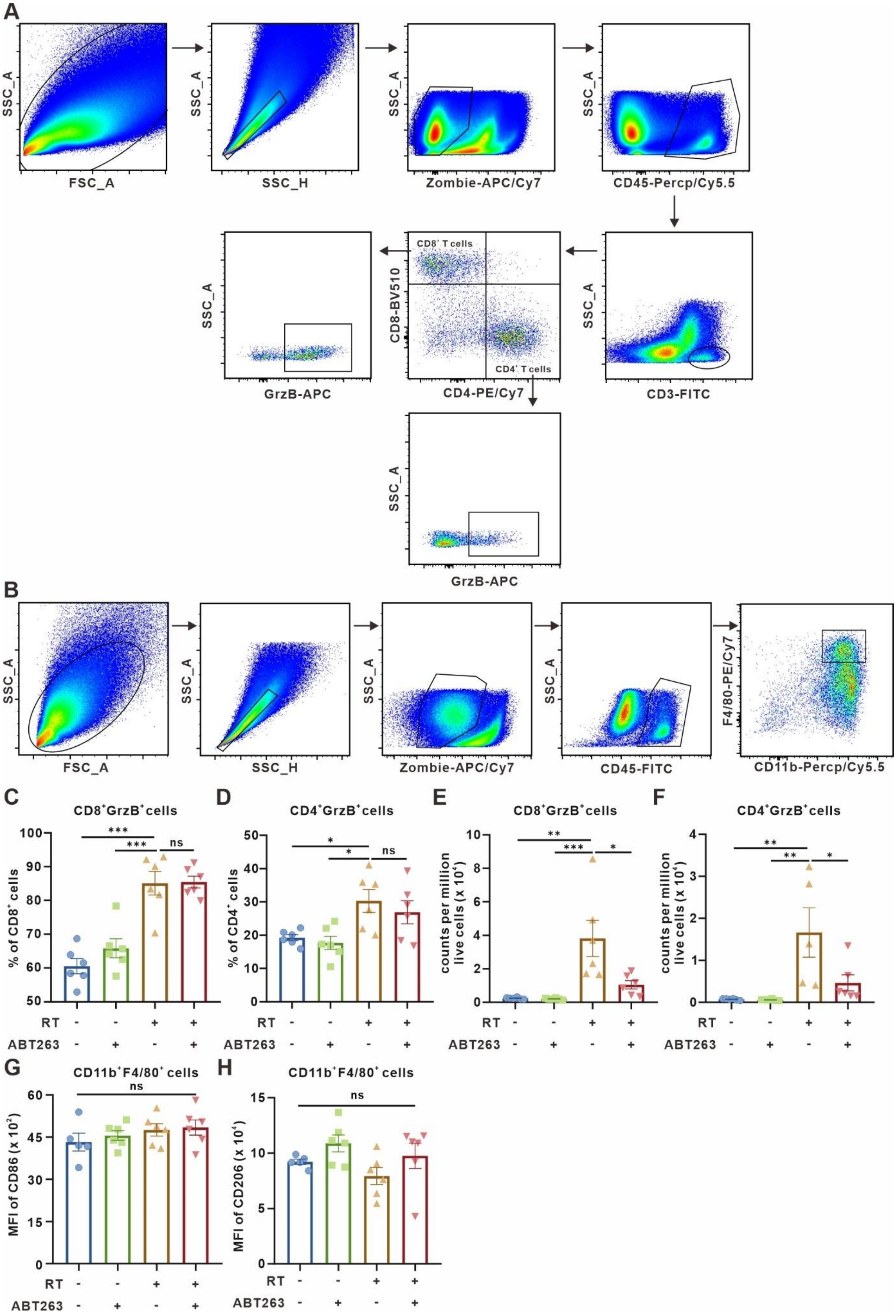
A
SSC_A
FSC_A
SSC_H
Zombie-APC/Cy7
CD45-Percp/Cy5.5
CD8⁺ T cells
CD8-BV510
CD4⁺ T cells
CD4-PE/Cy7
CD3-FITC
GrzB-APC
B
CD45-FITC
F4/80-PE/Cy7
CD11b-Percp/Cy5.5
C
CD8⁺GrzB⁺cells
% of CD8⁺ cells
D
CD4⁺GrzB⁺cells
% of CD4⁺ cells
E
CD8⁺GrzB⁺cells
counts per million live cells (x 10⁴)
F
CD4⁺GrzB⁺cells
counts per million live cells (x 10⁴)
G
CD11b⁺F4/80⁺ cells
MFI of CD86 (x 10²)
H
CD11b⁺F4/80⁺ cells
MFI of CD206 (x 10⁴)
RT
ABT263
ns

Figure S6. Effects of senescence removal treatment on the immune microenvironment.

(A) Gating strategy for detection of the $CD4^+$ T cells and $CD8^+$ T cells by flow cytometry. (B) Gating strategy for detection of the TAMs by flow cytometry. (C-D) Flow cytometry analysis of changes in the $CD8^+GrzB^+$ T (C) cells and $CD4^+GrzB^+$ T cells (D) in the TME of LLC subcutaneous transplant model that underwent different treatments (n = 6 per group). (E-F) Flow cytometry analysis of changes in the absolute counts of $CD8^+GrzB^+$ T (E) cells and $CD4^+GrzB^+$ T cells (F) in the TME of LLC subcutaneous transplant model that underwent different treatments (n = 5 to 6 per group). (G-H) Flow cytometric analysis of CD86 (G) and CD206 (H) expression in TAMs of LLC subcutaneous transplant model that underwent different treatments (n = 5 to 6 per group). The indicated results represent the mean ± SEM, analyzed by one-way ANOVA. *$p < 0.05$; **$p < 0.01$; ***$p < 0.001$; ns, not statistically significant.

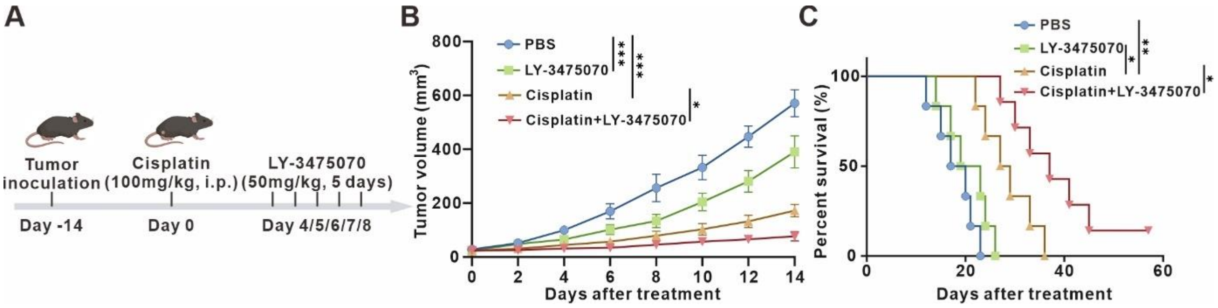

Figure S7. Cisplatin chemotherapy-induced senescence combined with CD73 inhibitor suppresses tumor growth.

(A) Schematic of experiment to assess efficacy of LY-3475070 in the cisplatin chemotherapy-induced senescence mouse model. (B) Tumor growth curves of LLC subcutaneous transplant model in corresponding treatment groups (n = 6 to 7 per group), analyzed by 2-way ANOVA. (C) Kaplan-Meier survival plot of LLC lung cancer-bearing mice in the corresponding treatment groups described in (B) (n = 6 to 7 per group). *$p < 0.05$; **$p < 0.01$; ***$p < 0.001$.

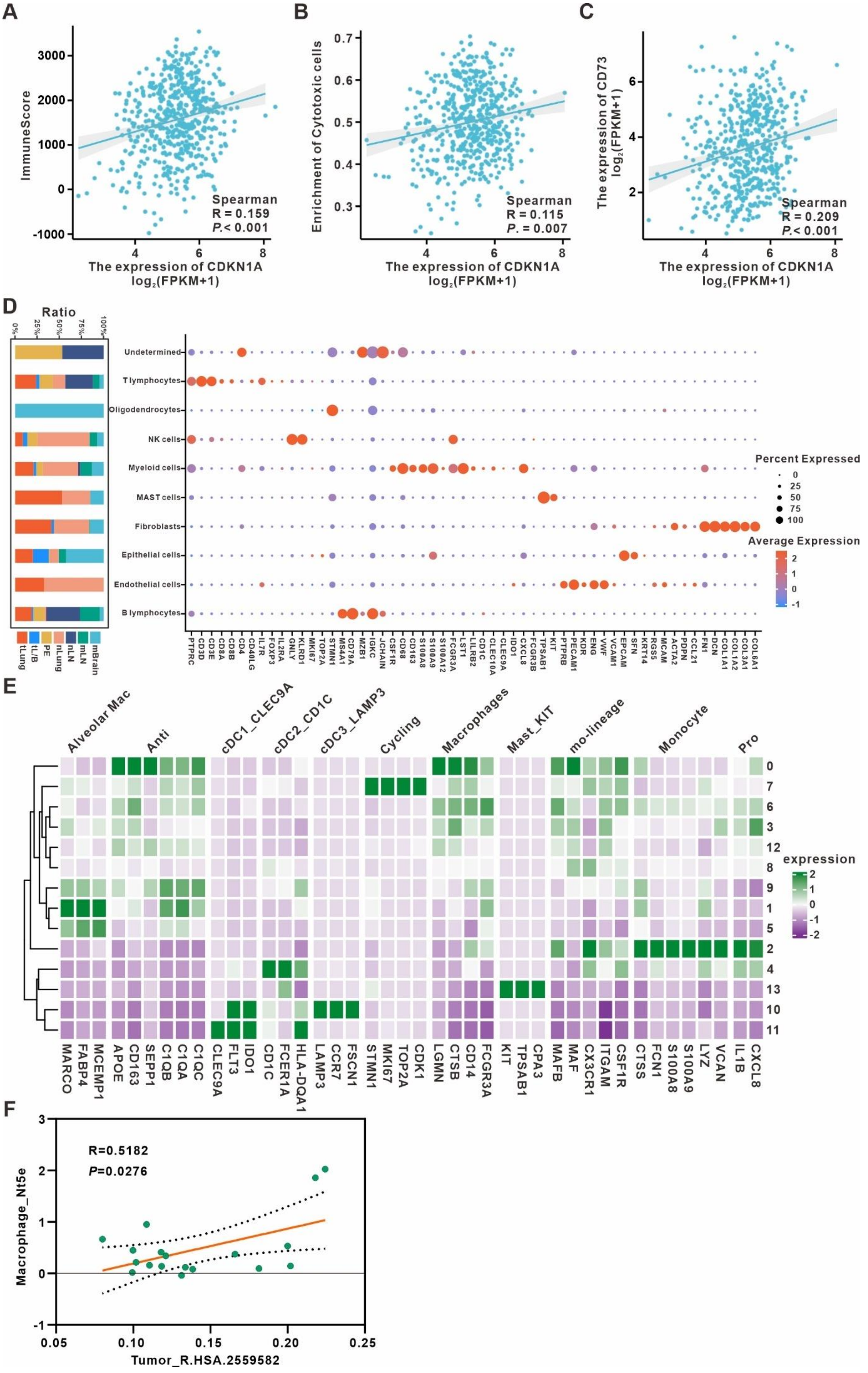
A
ImmuneScore
Spearman
R = 0.159
P.< 0.001
The expression of CDKN1A
log2(FPKM+1)
B
Enrichment of Cytotoxic cells
Spearman
R = 0.115
P. = 0.007
The expression of CDKN1A
log2(FPKM+1)
C
The expression of CD73
log2(FPKM+1)
Spearman
R = 0.209
P.< 0.001
The expression of CDKN1A
log2(FPKM+1)
D
Ratio
Undetermined
T lymphocytes
Oligodendrocytes
NK cells
Myeloid cells
MAST cells
Fibroblasts
Epithelial cells
Endothelial cells
B lymphocytes
tLung
tL/B
PE
nLung
nLN
mLN
mBrain
Percent Expressed
Average Expression
E
Alveolar Mac
Anti
cDC1_CLEC9A
cDC2_CD1C
cDC3_LAMP3
Cycling
Macrophages
Mast_KIT
mo-lineage
Monocyte
Pro
expression
MARCO
FABP4
MCEMP1
APOE
CD163
SEPP1
C1QB
C1QA
C1QC
CLEC9A
FLT3
IDO1
CD1C
FCER1A
HLA-DQA1
LAMP3
CCR7
FSCN1
STMN1
MKI67
TOP2A
CDK1
LGMN
CTSB
CD14
FCGR3A
KIT
TPSAB1
CPA3
MAFB
MAF
CX3CR1
ITGAM
CSF1R
CTSS
FCN1
S100A8
S100A9
LYZ
VCAN
IL1B
CXCL8
F
R=0.5182
P=0.0276
Macrophage_Nt5e
Tumor_R.HSA.2559582

100

Figure S8. Bioinformatics analysis to verify the correlation between senescence and macrophage CD73 expression.

(A) Association between CDKN1A expression and immune score in TCGA lung adenocarcinoma dataset. (B) Association between CDKN1A expression and cytotoxic cells enrichment in TCGA lung adenocarcinoma dataset. (C) The expression correlation between CDKN1A and CD73 in TCGA lung adenocarcinoma dataset. (D) Left panel, tissue preference of each cluster. Right panel, dot plot of mean expression of canonical marker genes for ten major lineages from tissues of each origin, as indicated. (E) Heatmap of selected myeloid cell marker genes in each cell cluster. (F) Linear regression analysis between the SASP gene set (R.HSA.2559582) expression of tumor cells and the CD73 expression of macrophages in the single-cell dataset.

Table S1. Sequences of designed siRNA targeting STAT3.

siRNA	SenseSeq (5'-3')	Seq (5'-3')
#1	CGACUUUGAUUUCAACUACAA	UUGUAGUUGAAAUCAAAGUCG
#2	CCUGAGUUGAAUUAUCAGCUU	AAGCUGAUAAUUCAACUCAGG
#3	CACCAUUCAUUGAUGCAGUUU	AAACUGCAUCAAUGAAUGGUG

Table S2. Sequences of designed shRNA targeting p65.

shRNA	Sequence (5’-3’)	Company
#1	GGAGTACCCTGAAGCTATAAC	Vigene Biology
#2	CTGTCCTCTCACATCCGATTT	Vigene Biology

Table S3. Antibodies used in this article.

Antibodies	Source	Detail information
GAPDH	ABclonal	GAPDH Rabbit pAb (AC001)
CDKN1A	Proteintech	P21 Polyclonal antibody #10355-1-AP
STAT3	Proteintech	STAT3 Polyclonal antibody #10253-2-AP
p-STAT3	CST	Phospho-Stat3 (Tyr705) (D3A7) XP® Rabbit mAb #9145
Second antibody	ABclonal	HRP Goat Anti-Rabbit IgG (H+L) (AS014)
Second antibody	ABclonal	HRP Goat Anti-Mouse IgG (H+L) (AS003)

Table S4. Sequences of primers for RT-qPCR analysis.

Gene name	Forward Primer	Reverse Primer
Cdkn1a	CCTGGTGATGTCCGACCTG	CCATGAGCGCATCGCAATC
Il6	TAGTCCTTCCTACCCCAATTTCC	TTGGTCCTTAGCCACTCCTTC
Gapdh	TGACCTCAACTACATGGTCTACA	CTTCCCATTCTCGGCCTTG
Cd39	AAGGTGAAGAGATTTTGCTCCAA	TTTGTTCTGGGTCAGTCCCAC
Cd73	GGACATTTGACCTCGTCCAAT	GGGCACTCGACACTTGGTG
Rela	AGGCTTCTGGGCCTTATGTG	TGCTTCTCTCGCCAGGAATAC
Il7	GCTGCTTTTCTAAATCGTGCTGCTC	GTTCACCAGTGTTTGTGTGCCTTG
Il8	TCGGGAGACCTCTAGACACTTTGC	GCCTGTCAAGCTGACTTCACTGG
Csf1	CCAATGCTAACGCCACCGAGAG	GCCTTGTTCTGCTCCTCATAGTCC
Csf2	GCATTGTGGTCTACAGCCTCTCAG	GGCATGTCATCCAGGAGGTTCAG
Csf3	TGCCAACTTTGCCACCACCATC	CGCTGGAAGGCAGAAGTGAAGG
Icam1	TCCGCTGTGCTTTGAGAACTGTG	AGGGTGAGGTCCTTGCCTACTTG
Tnfa	CGCTCTTCTGTCTACTGAACTTCGG	GTGGTTTGTGAGTGTGAGGGTCTG
Il1b	GCAACTGTTCCTGAACTCAACT	ATCTTTTGGGGTCCGTCAACT
Cxcl1	GGCTGGGATTCACCTCAAGAACATC	TGAGTGTGGCTATGACTTCGGTTTG
Cxcl2	AACATCCAGAGCTTGAGTGTGACG	GGGCTTCAGGGTCAAGGCAAAC
Cxcl3	GCCACTCTCAAGGATGGTCAAGAAG	GGACTTGCCGCTCTTCAGTATCTTC
Cxcl5	TGCGTTGTGTTTGCTTAACCGTAAC	TGACTTCCACCGTAGGGCACTG
Cxcl9	TCCTTTTGGGCATCATCTTCC	TTTGTAGTGGATCGTGCCTCG
Cxcl10	CCAAGTGCTGCCGTCATTTTC	GGCTCGCAGGGATGATTTCAA
Ccl2	CACTCACCTGCTGCTACTCATTCAC	CTTCTTTGGGACACCTGCTGCTG
Ccl3	CTCCCAGCCAGGTGTCATTTTCC	CAGGCATTCAGTTCCAGGTCAGTG
Ccl5	GACACCACTCCCTGCTGCTTTG	CTCTGGGTTGGCACACACTTGG
Ccl7	GCTTCTGTGCCTGCTGCTCATAG	GCTCTTGAGATTCCTCTTGGGGATC
Ccl8	GCTCCAGTCACCTGCTGCTTTC	ACACAGAGAGACATACCCTGCTTGG
Ccl11	CTATTCCTGCTGCTCACGGTCAC	GCTCTTCAGTAGTGTGTTGGGGATC
Ccl20	AGGCAGAAGCAAGCAACTACGAC	ATCGGCCATCTGTCTTGTGAAACC
Mmp2	ACCATGCGGAAGCCAAGATGTG	AGGGTCCAGGTCAGGTGTGTAAC
Mmp3	GACGATGATGAACGATGGACAGAGG	TGTGGAGGACTTGTAGACTGGGTAC
Ccl19	TGCTAATGATGCGGAAGACTGCTG	TCCTTCTGGTGCTGTTGCCTTTG
Ccl1	GCCGTGTGGATACAGGATGTTGAC	TAGCAGGGGTTCACCTTCTTCAGG
Ccl17	GGTCACTTCAGATGCTGCTCCTG	CCTGGACAGTCAGAAACACGATGG
Ccl21a	GAGCCTCCTTAGCCTGGTCCTG	TGTTCAGTTCTCTTGCAGCCCTTG
Ccl22	CTCCTGGTGGCTCTCGTCCTTC	CGGCACAGATATCTCGGTTCTTGAC

Gene name	Forward Primer	Reverse Primer
Ccl25	TTTGAAGACTGCTGCCTGGGTTAC	CACTCCTCACGCTTGTACTGTTGG
Ccl27a	CTGGCATCCGTGGAACAAGACTAAG	GTCCCTTGGAGCCTTTTCCCTTG
Cx3cl1	TGGAAGACCTTGCTTTGGAACTGAC	AAGTAGTGGACACCTGAGGAGATGG
Ccl27	CTACCGAAAGCCACTCTCAGACAAG	TCAGCCCATTTTCCTTAGCATCCC
Ccl28	GTGTGTGTGGCTTTTCAAACCTCAG	AAGTACGATTGTGCGGGCTGATG
Stat3	CAATACCATTGACCTGCCGAT	GAGCGACTCAAACTGCCCT
